# m6A-enriched lncRNA *LINC00839* promotes tumor progression by enhancing TAF15-mediated transcription of amine oxidase *AOC1* in nasopharyngeal carcinoma

**DOI:** 10.1016/j.jbc.2023.104873

**Published:** 2023-05-29

**Authors:** Wei-Hong Zheng, Zhi-Qing Long, Zi-Qi Zheng, Lu-Lu Zhang, Ye-Lin Liang, Zhi-Xuan Li, Jia-Wei Lv, Jia Kou, Xiao-Hong Hong, Shi-Wei He, Rui Xu, Guan-Qun Zhou, Na Liu, Jun Ma, Ying Sun, Li Lin, Denghui Wei

**Affiliations:** 1 Department of Radiation Oncology, Sun Yat-sen University Cancer Center, State Key Laboratory of Oncology in South China, Collaborative Innovation Center for Cancer Medicine, Guangdong Key Laboratory of Nasopharyngeal Carcinoma Diagnosis and Therapy, Guangzhou, China; 2 State Key Laboratory of Oncology in Southern China, Collaborative Innovation Center for Cancer Medicine, Sun Yat-sen University Cancer Center, Guangzhou, China; 3 Department of Molecular Diagnostics, Sun Yat-sen University Cancer Center, State Key Laboratory of Oncology in South China, Collaborative Innovation Center for Cancer Medicine, Guangdong Key Laboratory of Nasopharyngeal Carcinoma Diagnosis and Therapy, Guangzhou, China

**Keywords:** nasopharyngeal carcinoma, progression, *LINC00839*, m6A, *TAF15*

## Abstract

Dysregulation of long noncoding RNAs (lncRNAs) contributes to tumorigenesis by modulating specific cancer-related pathways, but the roles of N6-methyladenosine (m6A)-enriched lncRNAs and underlying mechanisms remain elusive in nasopharyngeal carcinoma (NPC). Here, we reanalyzed the previous genome-wide analysis of lncRNA profiles in 18 pairs of NPC and normal tissues as well as in ten paired samples from NPC with or without post-treatment metastases. We discerned that an oncogenic m6A-enriched lncRNA, *LINC00839*, which was substantially upregulated in NPC and correlated with poor clinical prognosis, promoted NPC growth and metastasis both *in vitro* and *in vivo*. Mechanistically, by using RNA pull-down assay combined with mass spectrometry, we found that *LINC00839* interacted directly with the transcription factor, TATA-box binding protein associated factor (TAF15). Besides, chromatin immunoprecipitation and dual-luciferase report assays demonstrated that *LINC00839* coordinated the recruitment of TAF15 to the promoter region of amine oxidase copper-containing 1 (*AOC1*), which encodes a secreted glycoprotein playing vital roles in various cancers, thereby activating *AOC1* transcription in *trans*. In this study, potential effects of *AOC1* in NPC progression were first proposed. Moreover, ectopic expression of *AOC1* partially rescued the inhibitory effect of downregulation of *LINC00839* in NPC. Furthermore, we showed that silencing vir-like m6A methyltransferase-associated (*VIRMA*) and insulin-like growth factor 2 mRNA-binding proteins 1 (*IGF2BP1*) attenuated the expression level and RNA stability of *LINC00839* in an m6A-dependent manner. Taken together, our study unveils a novel oncogenic VIRMA/IGF2BP1–*LINC00839*–TAF15–AOC1 axis and highlights the significance and prognostic value of *LINC00839* expression in NPC carcinogenesis.

Nasopharyngeal carcinoma (NPC) is a head and neck squamous cell tumor endemic in Southeastern Asia and North Africa (1). Radiotherapy is the mainstay treatment modality for NPC because of its specific anatomic and biological characteristics (2, 3, 4). With the development of intensity-modulated radiotherapy technique and combined strategy of chemotherapy, survival outcomes of NPC have been greatly improved during the past decades (5). However, approximately 30% of the NPC patients still relapse with locoregionally recurrence and/or distant metastasis (6, 7, 8). Therefore, identification of prognostic biomarkers together with elucidation of relevant molecular mechanisms is warranted for risk stratification of NPC patients and establishment of individualized treatment.

Long noncoding RNAs (lncRNAs), defined as transcripts longer than 200 nucleotides that are not translated into functional proteins, have been proposed as regulators of diverse fundamental biological processes. Accumulating researches exemplify that dysregulations of lncRNAs contribute to tumorigenesis and progression by modulating specific cancer-related pathways (9, 10, 11). Mechanistically, lncRNAs could directly bind to DNA, RNA, or protein, function as scaffold or guide molecule to protein–protein or protein–nucleotide interactions, and regulate multiple targets at transcriptional or post-transcriptional levels. Among the most prominent proposed functions of lncRNAs is the establishment of an active or a repressive chromatin state (12, 13). With respect to NPC, aberrant lncRNA expression profiles have been reported (14, 15), and several lncRNAs, like *DANCR*, *TINCR*, etc, were proved to be involved in cancer-related mechanism (16, 17). Nevertheless, the exact function and biological relevance of the vast majority of lncRNAs remain unclear.

Mounting evidence indicates that various post-transcriptional modifications of RNAs, such as N6-methyladenosine (m6A) and 5-methylcytosine, participate in modulation of transcript stability, alternative splicing, and subcellular localization (18, 19). Among them, m6A is the most predominant modification with a consensus sequence of RRACH (R corresponds to G or A; H corresponds to A, C, or U). This dynamic and reversible epigenetic regulatory process relies on m6A methyltransferase complex (writer), demethylases (eraser), and reader proteins (reader) (20). It has been reported that m6A modification plays vital roles in NPC progression (21, 22, 23, 24). Recent research has suggested that Wilms tumor 1–associated protein (WTAP) promotes NPC proliferation and metastasis by mediating m6A methylation status of lncRNA *DIAPH1-AS1*. However, the functions of m6A-enriched lncRNA and its underlying mechanisms are still unclear in NPC (24).

In this study, by reanalyzing the lncRNA expression profile (25), we discerned that an oncogenic m6A-enriched lncRNA, *LINC00839*, which was substantially upregulated in NPC and correlated with poor clinical prognosis. *LINC00839* is situated on10q11.21 encoding long intergenic noncoding RNA, which has five exons and is mainly transcribed into a transcript of 2322 nt. Previous studies have proposed that *LINC00839* is responsible for multiple cancer biological traits, including tumor growth, metastasis, and chemoresistance (26, 27, 28, 29). *LINC00839* is identified targeting specific tumor signaling pathways and exacerbates malignant features of several tumors, such as breast cancer (26) and osteoblastoma (28). Here, we found that *LINC00839* promoted NPC proliferation and metastasis by mediating the recruitment of transcriptional factor TATA-box binding protein associated factor (TAF15) to the promoter region of amine oxidase copper-containing 1 (*AOC1*) and activating *AOC1* transcription in *trans*. Furthermore, vir like m6A methyltransferase associated (VIRMA) and insulin-like growth factor 2 mRNA-binding proteins 1 (IGF2BP1) were found to promote the stability of *LINC00839* in an m6A-dependent manner. This study identified a novel oncogenic VIRMA/IGF2BP1–*LINC00839*–TAF15–AOC1 axis and highlights the pivotal role and prognostic potential of *LINC00839* in NPC progression.

## Results

### *LINC00839* is overexpressed and correlates with poor prognosis in NPC

**Figure 1:**
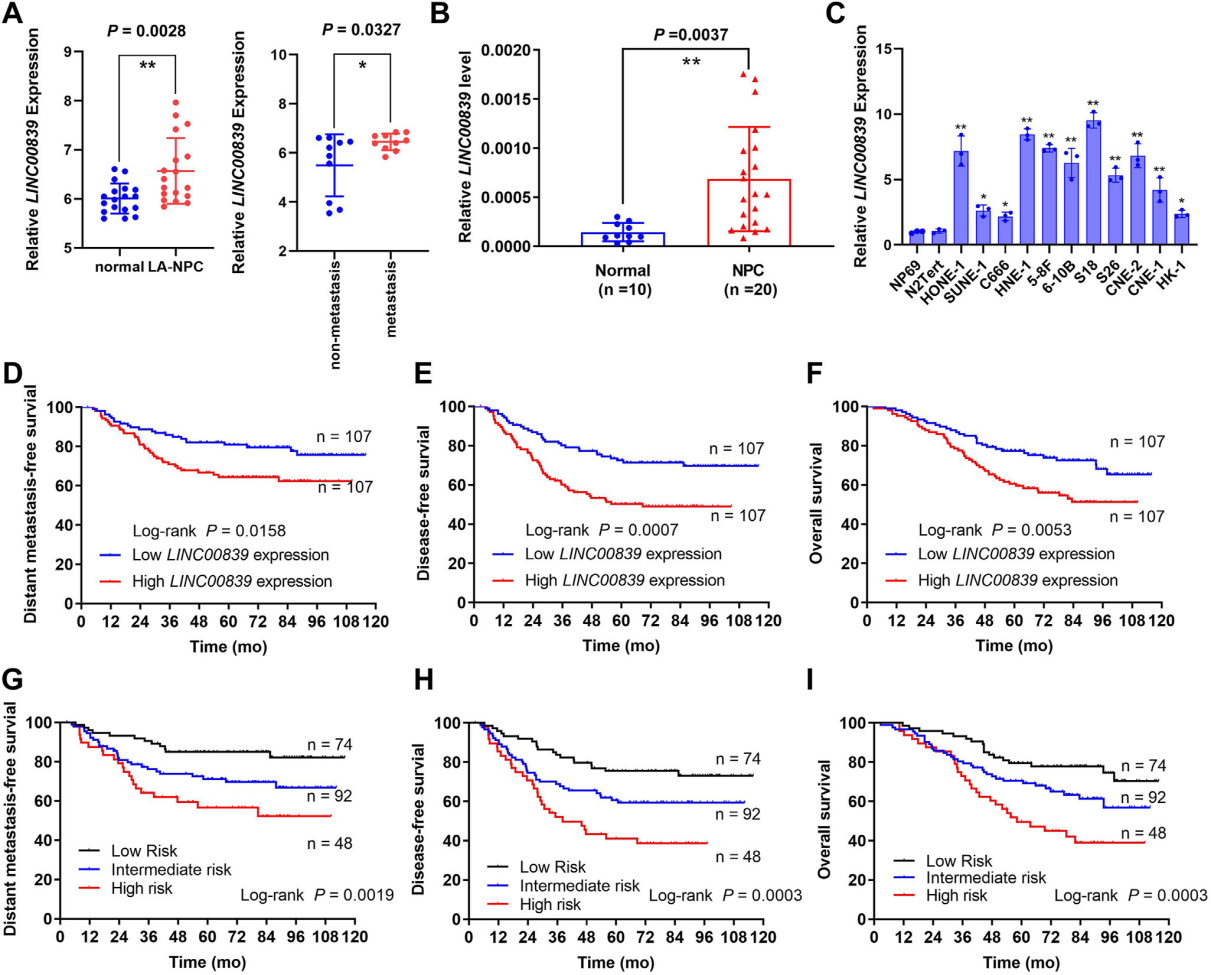
***LINC00839* is overexpressed and correlates with poor prognosis in NPC.***A*, *LINC00839* expression in 18 paired LA-NPC and normal tissues (*left*) and 10 paired samples from LA-NPC developed with or without distant metastases (*right*), based on previous microarray data. *B*, *LINC00839* expression in NPC (n = 20) and normal nasopharynx tissues (n = 10) detected by RT–qPCR. *C*, *LINC00839* expression in 11 NPC cell lines and two immortalized nasopharyngeal epithelial cells (NP69 and N2Tert). *D*–*I*, *LINC00839* expression in 214 paraffin-embedded NPC tissues. Kaplan–Meier curves of distant metastasis-free survival (*D*), disease-free survival (*E*), and overall survival (*F*) according to high (n = 107) or low (n = 107) *LINC00839* expression groups, which was divided by the median expression level of *LINC00839*. Kaplan–Meier analysis of distant metastasis-free survival (*G*), disease-free survival (*H*), and overall survival (*I*) according to the prognostic prediction model, low risk (low *LINC00839* expression and early TNM stage, n = 74), intermediate risk (high *LINC0839* expression or advanced TNM stage, n = 92), and high risk (high *LINC00839* expression and advanced TNM stage, n = 48). Data are presented as the mean ± SEM or mean ± SD. ∗*p* < 0.05, ∗∗*p* < 0.01. The experiments were repeated at least three times independently. NPC, nasopharyngeal carcinoma; qPCR, quantitative PCR.

To determine lncRNAs involved in NPC progression, we previously performed genome-wide analysis of lncRNA profile in 18 pairs of locoregionally advanced NPC (LA-NPC) tissues and matched healthy controls as well as ten paired samples from LA-NPC developed with or without post-treatment distant metastases (25). Through reanalyzing these microarray data, *LINC00839* was found to be highly expressed in LA-NPC (*p* = 0.0028, fold change = 1.47) *versus* normal nasopharynx tissues as well as in LA-NPC developed with metastasis (*p* = 0.0327, fold change = 1.94) *versus* nonmetastatic group (Fig. 1*A*). In addition, we detected the expression level of *LINC00839* in 20 freshly frozen NPC and 10 normal nasopharyngeal epithelial tissues using RT–quantitative PCR (qPCR) and found that *LINC00839* was significantly overexpressed in NPC tissues (Fig. 1*B*, *p* = 0.0037). Besides, *LINC00839* expression levels were remarkably upregulated in 11 NPC cell lines compared with two immortalized normal nasopharyngeal epithelial cell lines, NP69 and N2Tert (Fig. 1*C*, all *p* < 0.05). These results indicate that *LINC00839* may act as an oncogene in NPC.

**Supporting Figure S1:**
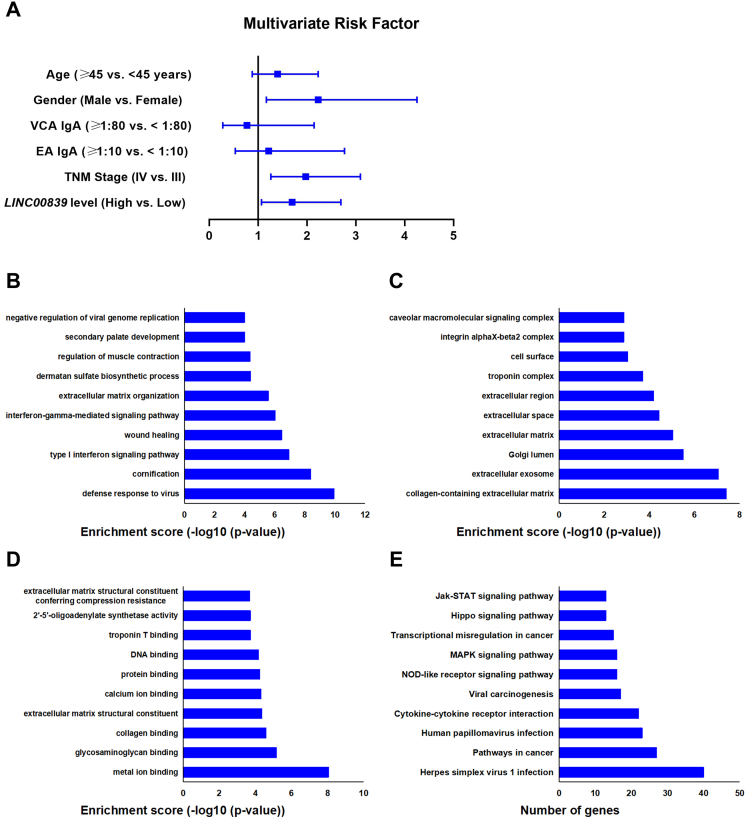
**Results and relevant bioinformatic analyses of *LINC00839* RNA-sequencing.***A*, Multivariate Cox regression analysis of clinical prognostic variables for OS of patients with NPC (n = 214). *B*–*E*, Gene Oncology (GO) analyses of biological processes (*B*), cellular components (*C*), and molecular functions (*D*); Kyoto Encyclopedia of Genes and Genomes (KEGG) analysis (*E*).

To further determine its prognostic value, we subsequently examined expression levels of *LINC00839* with 214 paraffin-embedded NPC tissues using RT–qPCR. Patients diagnosed with NPC were stratified into two groups (high *LINC00839* expression group *versus* low *LINC00839* expression group, n = 107) according to median expression. Kaplan–Meier survival analyses were conducted, and results demonstrated that patients with high *LINC00839* level had more disease failures as well as poorer prognoses (Fig. 1, *D*–*F*, all *p* < 0.05). Clinicopathological characteristics of patients are presented in Table S1. Multivariate Cox regression analyses revealed that both *LINC00839* expression level and TNM stage were independent prognostic factors of distant metastasis-free survival, disease-free survival, and overall survival for NPC patients (Fig. S1 and Table S2, all *p* < 0.05). Moreover, we combined *LINC00839* expression data and TNM stage to establish a prognostic stratification model and stratified NPC patients into three risk groups: low-risk group (low *LINC00839* expression and early TNM stage, n = 74), intermediate-risk group (high *LINC00839* expression or advanced TNM stage, n = 92), and high-risk group (high *LINC00839* expression and advanced TNM stage, n = 48). As expected, corresponding survival curves suggested that patients of these three risk groups had significantly different survival outcomes (Fig. 1, *G*–*I*, all *p* < 0.01).

### *LINC00839* facilitates NPC proliferation, migration, and invasion *in vitro*

**Figure 2:**
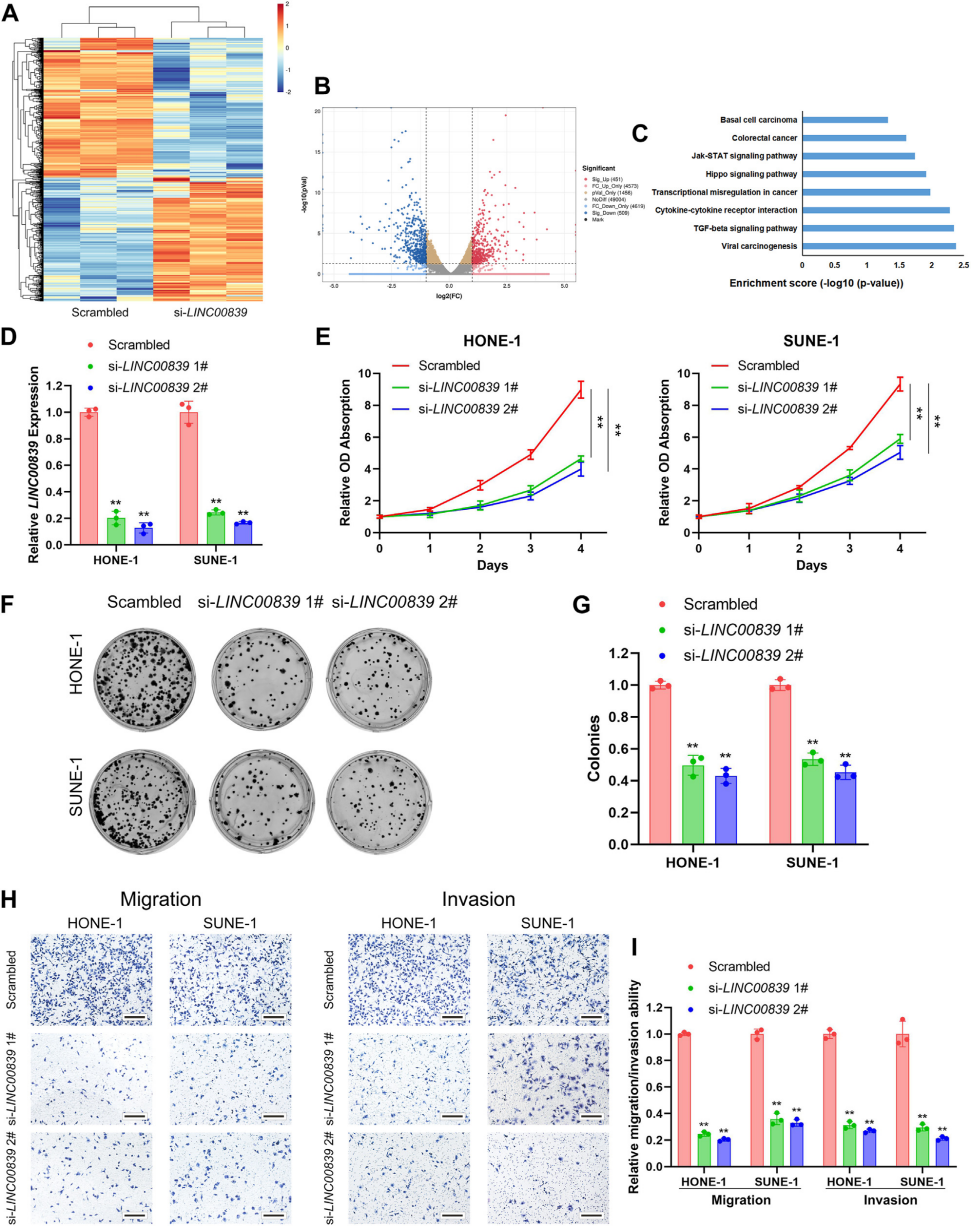
**Knockdown of *LINC00839* impairs NPC proliferation, migration, and invasion *in vitro*.***A* and *B*, heatmap (*A*) and scatter plot (*B*) of the differentially expressed genes between three pairs of SUNE-1 cells transfected with scrambled or si-*LINC00839* (|log_2_ fold change| >1 and *p* < 0.05). *C*, KEGG pathway analysis of genes regulated by *LINC00839* in SUNE-1 cells. Transcriptional misregulation in cancer was among the significant pathways. *D*, *LINC00839* expression in HONE-1 and SUNE-1 cells transfected with si-*LINC00839*s or the scrambled control. *E*, CCK-8 assays in HONE-1 and SUNE-1 cells transfected with si-*LINC00839*s or the scrambled control. *F* and *G*, representative images (*F*) and quantification (*G*) of colonies formed in HONE-1 and SUNE-1 cells transfected with si-*LINC00839*s or the scrambled control. *H* and *I*, representative images (*H*) and quantification (*I*) of migratory or invasive cells in HONE-1 and SUNE-1 cells transfected with si-*LINC00839*s or the scrambled control. Scale bars for plot (*H*) represent 200 μm. Data are presented as the mean ± SD. ∗*p* < 0.05, ∗∗*p* < 0.01. The experiments were repeated at least three times independently. CCK-8, Cell Counting Kit-8; KEGG, Kyoto Encyclopedia of Genes and Genomes; NPC, nasopharyngeal carcinoma.

We first performed RNA-Seq in a typical NPC cell line (SUNE-1 cells) transfected with scrambled control or si-*LINC00839* and identified 451 upregulated genes and 509 downregulated genes (|log_2_ fold change| >1 and *p* < 0.05, Fig. 2, *A* and *B*). Kyoto Encyclopedia of Genes and Genomes (KEGG) analysis revealed that genes affected by endogenous *LINC00839* silencing were enriched in cancer-related pathways, indicating a potential regulatory role of *LINC00839* in NPC progression (Fig. 2*C*).

**Supporting Figure S2:**
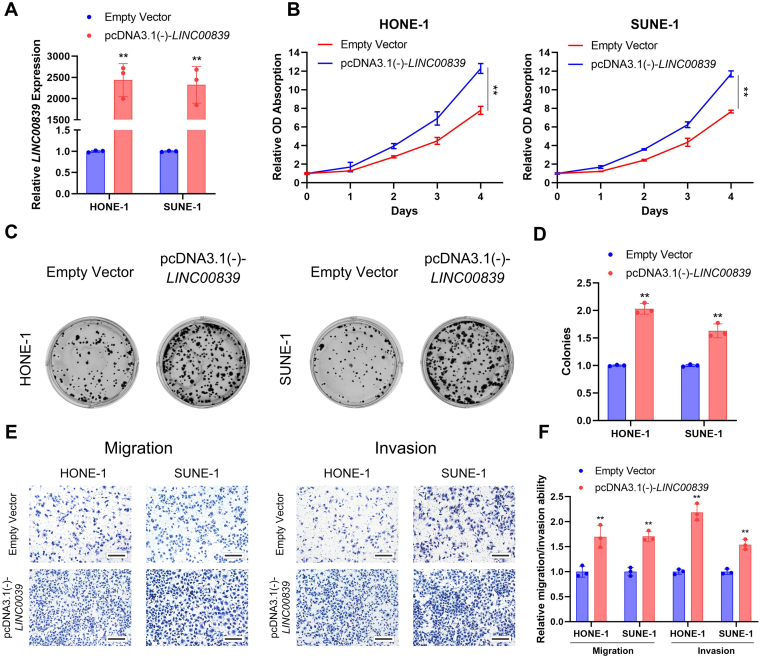
**Over-expression of *LINC00839* facilitates NPC proliferation, migration and invasion *in vitro*.***A*, *LINC00839* expression in HONE-1 and SUNE-1 cells transfected with pcDNA3.1(-)-*LINC00839* or empty vector. *B*, CCK8 assays in HONE-1 and SUNE-1 cells transfected with pcDNA3.1(-)-*LINC00839* or empty vector. *C* and *D*, Representative images (*C*) and quantification (*D*) of colonies formed in HONE-1 and SUNE-1 cells transfected with pcDNA3.1(-)-*LINC00839* or empty vector. *E* and *F*, Representative images (*E*) and quantification (*F*) of migratory or invasive cells in HONE-1 and SUNE-1 cells transfected with pcDNA3.1(-)-*LINC00839* or empty vector. Scale bars for plot (*E*): 200 μm. Data are presented as the mean ± SD. ∗*P* < 0.05, ∗∗*P* < 0.01. The experiments were repeated at least three times independently.

In order to validate the results, we transiently knocked down *LINC00839* expression in two NPC cell lines HONE-1 and SUNE-1 cells with two different siRNAs (Fig. 2*D*, *p* < 0.01). Cell Counting Kit-8 (CCK-8) and colony-formation assays were conducted, and we found that the knockdown of *LINC00839* significantly impaired the proliferation capacity of NPC cells *in vitro* (Fig. 2, *E*–*G*, all *p* < 0.01). In addition, Transwell assays showed that silencing *LINC00839* substantially suppressed the migration and invasion abilities of NPC cells (Fig. 2, *H* and *I*, all *p* < 0.01). On the contrary, the proliferation, migration, and invasion activities of NPC cells were facilitated by overexpression of *LINC00839 via* lentivirus infection (Fig. S2, all *p* < 0.01). Collectively, these findings suggested that *LINC00839* promoted proliferation, migration, and invasion of NPC cells *in vitro*.

### *LINC00839* directly binds and interacts with TAF15

**Figure 3:**
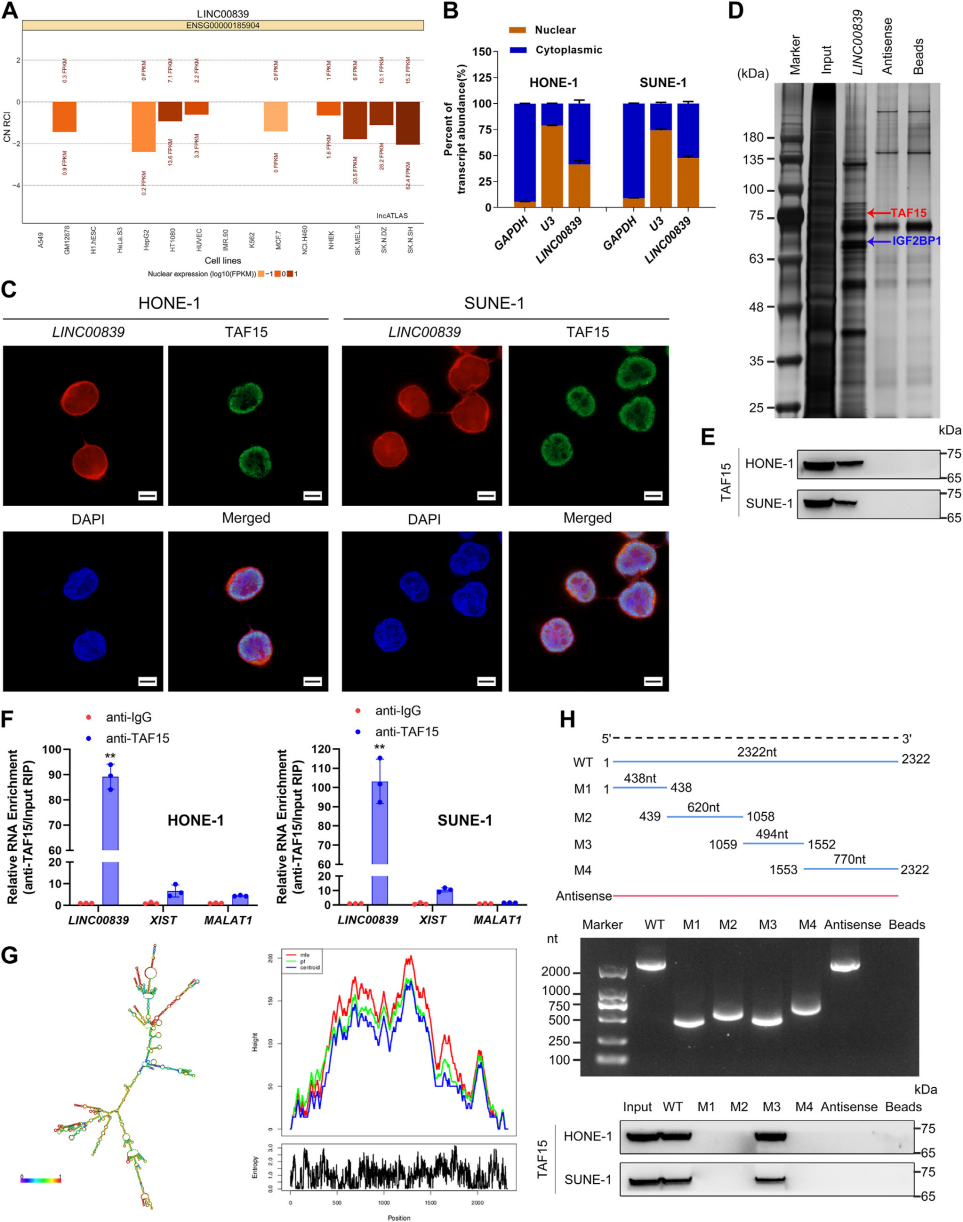
***LINC00839* directly interacts with TAF15 to promote NPC progression.***A*, bioinformatics tools of lncATLAS predicting that *LINC00839* is mainly located in the nucleus. *B*, subcellular *LINC00839* expression in the nucleus and cytoplasm of HONE-1 and SUNE-1 cells. *C*, RNA-FISH and immunofluorescence (IF) assays showing that *LINC00839* (Cy3; *red*) and TAF15 (*green*) are mainly colocalized in the nuclei. Nuclei, *blue* (DAPI). Scale bars for plot (*C*) represent 10 μm. *D*, RNA pull-down and silver staining of biotinylated *LINC00839*-associated proteins. Specific bands that were excised for mass spectrometry. The *arrows* indicate TAF15 (*red*) and IGF2BP1 (*blue*) proteins as the unique bands for *LINC00839*. *E*, *LINC00839* interacting with TAF15 as monitored by RNA pull-down and Western blotting analysis. *F*, *LINC00839* enrichment in the immunoprecipitated complexes using anti-TAF15 antibodies detected by RIP assay. *G*, the secondary structure (*left*) and mountain plot (*right*) of *LINC00839* was predicted using RNAfold WebServer. Minimum free energy (MFE), *red*; thermodynamic ensemble, *green*; centroid, *blue*. *H*, deletion mapping of the TAF15-binding domain in *LINC00839*. *Top*, diagrams of full-length *LINC00839* and its deletion fragments. *Middle*, the *in vitro* transcribed full-length *LINC00839* and truncated fragments with the correct sizes are indicated. *Bottom*, Western blotting analysis for TAF15 pulled down by truncated *LINC00839* transcripts. Data are presented as the mean ± SD. ∗*p* < 0.05, ∗∗*p* < 0.01. The experiments were repeated at least three times independently. DAPI, 6-diamidino-2-phenylindole; IGF2BP1, insulin-like growth factor 2 mRNA-binding protein 1; NPC, nasopharyngeal carcinoma; RIP, RNA immunoprecipitation; TAF15, TATA-box binding protein associated factor.

**Supporting Figure S3:**
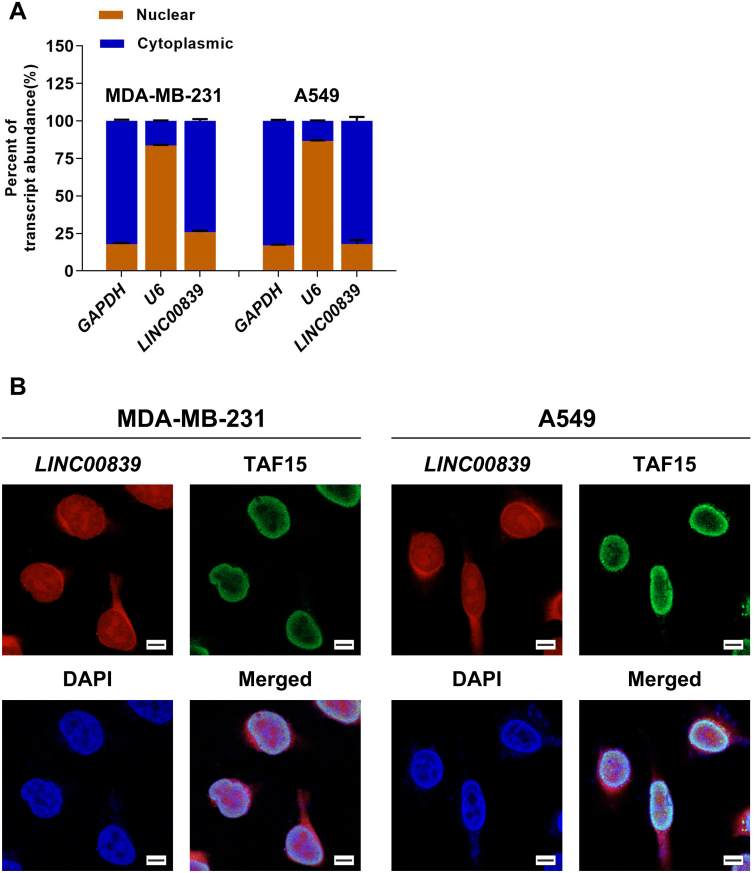
**Subcellular distribution of *LINC00839* in other cancer cells.***A*, Nucleocytoplasmic separation assays presenting the subcellular distribution of *LINC00839* in MDA-MB-231 and A549 cells. *B*, RNA-FISH and IF assays showing the distribution of *LINC00839* (Cy3; *red*) and TAF15 (*green*) in MDA-MB-231 and A549 cells. Nuclei, blue (DAPI). Scale bars for plot (*B*): 10 μm.

To characterize the molecular mechanism of *LINC00839* in NPC, we first determined the subcellular distribution of *LINC00839*. Prediction by lncATLAS (http://lncatlas.crg.eu/) indicated that *LINC00839* predominantly located in nucleus of all the available cell types (Fig. 3*A*). However, for NPC cells, nucleocytoplasmic separation and FISH assays suggested that *LINC00839* was located in both cytoplasm and nucleus of HONE-1 and SUNE-1 cells (Fig. 3, *B* and *C*). Moreover, distributions of *LINC00839* in another two cancer cell lines (MDA-MB-231 and A549 cells) were found more predominant in cytoplasm than that in nucleus (Fig. S3). LncRNAs have been reported to exert their biological functions by directly interacting with specific proteins. Therefore, an RNA pull-down assay followed by mass spectrometry (MS) was implemented to identify specific proteins binding to *LINC00839* (Fig. 3*D*). Among the highly enriched proteins, TAF15, a known transcription factor and RNA-binding protein, was identified. Its interaction with *LINC00839* was then verified by RNA pull-down and Western blotting analysis (Fig. 3*E*). To further confirm the physical interaction between *LINC00839* and TAF15, we performed an RNA immunoprecipitation (RIP) assay and found significant enrichment of *LINC00839* using anti-TAF15 antibodies rather than using immunoglobulin G (Fig. 3*F*, *p* < 0.01). FISH combined with immunofluorescence staining revealed that *LINC00839* was colocalized with TAF15 in the nucleus (Fig. 3*C*).

To further clarify the binding regions between *LINC00839* and TAF15, we first predicted the secondary structure and minimum free energy structure of *LINC00839* utilizing RNAfold WebServer (http://rna.tbi.univie.ac.at/) (Fig. 3*G*). Then, RNA pull-down assays using truncated *LINC00839* transcripts combined with Western blotting were performed, and the results suggested that 1059- to 1552-nt region of *LINC00839* was indispensable for interaction with TAF15 (Fig. 3*H*).

### *LINC00839* recruits TAF15 to activate *AOC1* transcription

**Figure 4:**
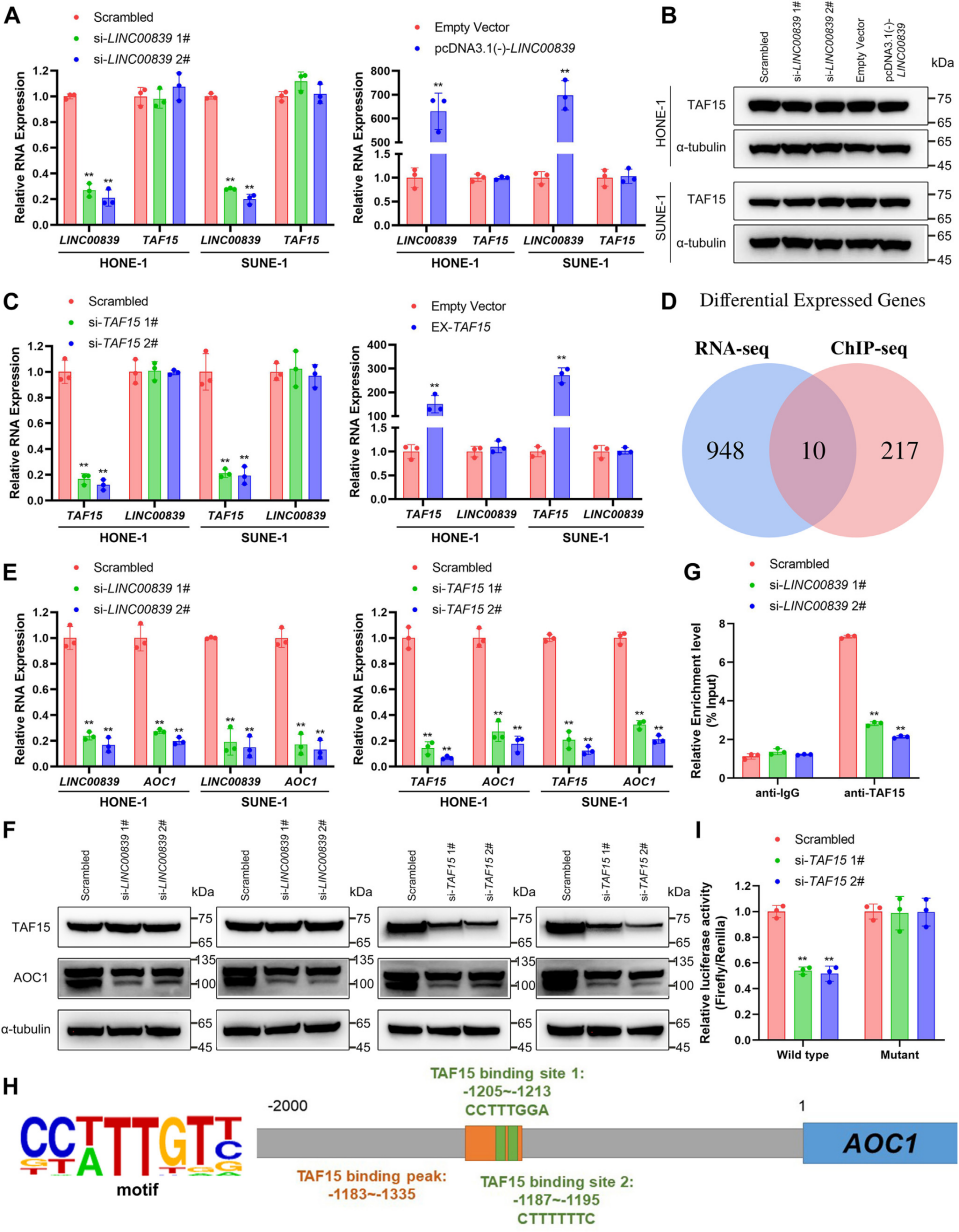
***LINC00839* recruits TAF15 to activate *AOC1* transcription.***A*, RNA expression level of *TAF15* in HONE-1 and SUNE-1 cells transfected with si-*LINC00839*s or the scrambled control (*left*) and transfected with pcDNA3.1(−)-*LINC00839* or empty vector (*right*). *B*, protein expression level of TAF15 in HONE-1 and SUNE-1 cells transfected with si-*LINC00839*s or the scrambled control (*left*) and transfected with pcDNA3.1(−)-*LINC00839* or empty vector (*right*). *C*, *LINC00839* expression in HONE-1 and SUNE-1 cells transfected with si-*TAF15*s or the scrambled control (*left*) and transfected with pUC57-*TAF15* or empty vector (*right*). *D*, overall analysis of RNA-Seq and ChIP-Seq data in the indicated cell lines. Overlapping gene sets identified among the differentially expressed genes after silencing of *LINC00839* (*blue*) and differently enriched genes in the TAF15 ChIP assay of HONE-1 cells with *LINC00839* silencing (*red*). *E*, RNA expression level of *AOC1* in HONE-1 and SUNE-1 cells transfected with si-*LINC00839*s or the scrambled control (*left*) and transfected with si-*TAF15*s or the scrambled control (*right*). *F*, protein expression level of AOC1 in HONE-1 and SUNE-1 cells transfected with si-*LINC00839*s or the scrambled control (*left*) and transfected with si-*TAF15*s or the scrambled control (*right*). *G*, silencing *LINC00839* decreased the enrichment of TAF15 on the *AOC1* promoter region in HONE-1 cells as determined by ChIP-qPCR assays. *H*, predicted binding sequences and schematics of the 5′ region (−2000 to 0 nt) of the *AOC1*. There are two TAF15-binding sites in the predicted TAF15-binding peak in promoter region of *AOC1*, −1205 to −1213 nt, and −1187 to −1195 nt. *I*, knockdown of *TAF15* inhibited the luciferase activity of the *AOC1* wildtype promoter construct rather than the mutant reporter gene construct, as indicated by luciferase reporter assays in HONE-1 cells. Data are presented as the mean ± SD. ∗*p* < 0.05, ∗∗*p* < 0.01. The experiments were repeated at least three times independently. *AOC1*, amine oxidase copper-containing 1; ChIP, chromatin immunoprecipitation; qPCR, quantitative PCR; TAF15, TATA-box binding protein associated factor.

**Supporting Figure S4:**
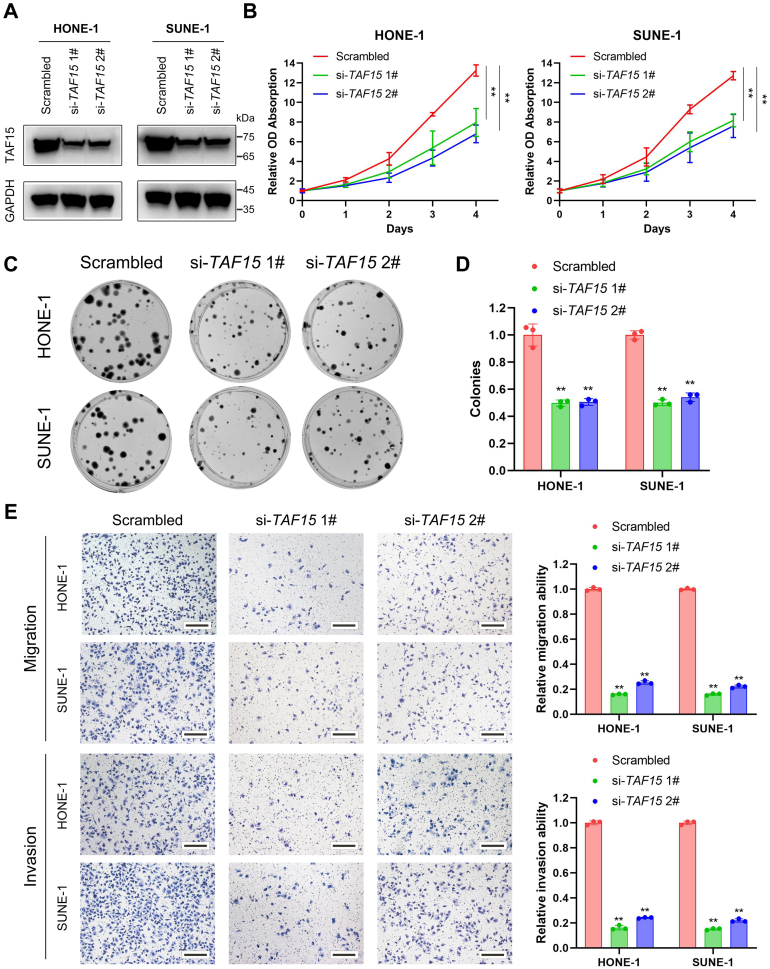
**TAF15 facilitates NPC proliferation, migration and invasion *in vitro*.***A*, TAF15 protein expression in HONE-1 and SUNE-1 cells transfected with si-*TAF15*s or the scrambled control. *B*, CCK8 assays in HONE-1 and SUNE-1 cells transfected with si-*TAF15*s or the scrambled control. *C* and *D*, Representative images (*C*) and quantification (*D*) of colonies formed in HONE-1 and SUNE-1 cells transfected with si-*TAF15*s or the scrambled control. *E*, Representative images (*left*) and quantification (*right*) of migratory (*upper*) or invasive (*lower*) cells in HONE-1 and SUNE-1 cells transfected with si-*TAF15*s or the scrambled control. Scale bars for plot (*E*): 200 μm. Data are presented as the mean ± SD. ∗*P* < 0.05, ∗∗*P* < 0.01. The experiments were repeated at least three times independently.

TAF15 is a member of FET protein family, which displays a high translocation rate in sarcoma and promotes carcinogenesis (30). Relevant studies reveal that TAF15 can bind DNA and fulfills pivotal functions in transcription (31, 32). We found that downregulation or overexpression of *LINC00839* had no effects on the expression of *TAF15*, reflected at either mRNA or protein levels (Fig. 4, *A* and *B*). Besides, the expression of *LINC00839* was not influenced by *TAF15* either (Fig. 4*C*). Next, we determined the role of *TAF15* in NPC and found that knockdown of *TAF15* impaired proliferation, migration, and invasion ability of NPC cells (Fig. S4).

**Supporting Figure S5:**
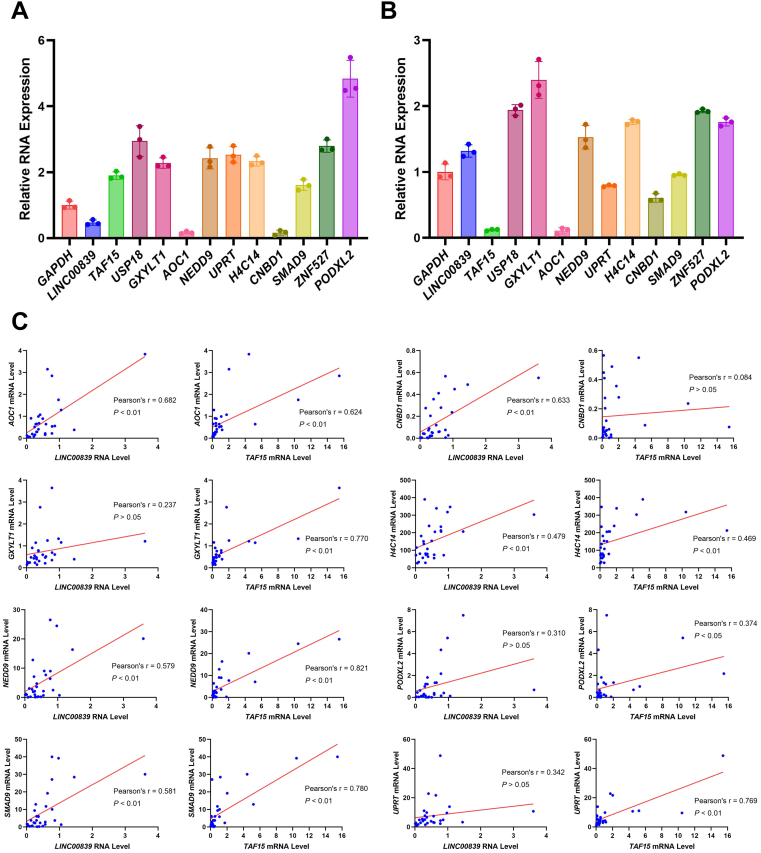
**RNA expressions and Pearson correlation analyses of overlapping genes.***A* and *B*, Results of ChIP-qPCR of overlapping genes with knockdown of *LINC00839* (*A*) and *TAF15* (*B*). *C*, Pearson correlation analyses between either *LINC00839* or *TAF15* and overlapping genes respectively in 30 NPC tissues. Data are presented as the mean ± SD. ∗*P* < 0.05, ∗∗*P* < 0.01. The experiments were repeated at least three times independently.

LncRNAs are known to play an oncogenic part by recruiting transcription factors to promoter regions of downstream oncogenes (9). To explore the potential regulatory mechanism of *LINC00839* and TAF15 in NPC, we conducted chromatin immunoprecipitation (ChIP) sequencing, and the results demonstrated that the knockdown of *LINC00839* effectively blocked TAF15 occupancy on certain target promoters. To recognize the target gene of *LINC00839*/TAF15 in NPC, we analyzed the RNA-Seq and ChIP-Seq data with or without *LINC00839* knockdown. We distinguished 10 genes, of which *AOC1* aroused our interest (Fig. 4*D*). The mRNA expression levels of *AOC1* were remarkably reduced when silencing *LINC00839* or *TAF15*, and Pearson correlation analyses revealed the significant correlations between either *LINC00839* or *TAF15* and *AOC1* in 30 NPC clinical specimens (Fig. S5).

AOC1 is a secreted glycoprotein that catalyzes the degradation of putrescine and histamine (33). Polyamines and related diamine precursor putrescine are found to be involved in cell growth and proliferation. Previous studies revealed that AOC1 played a vital role in gastric cancer and colorectal cancer progression (34, 35). We confirmed that the mRNA and protein expression of *AOC1* were significantly downregulated when knocking down *LINC00839* or *TAF15* using RT–qPCR and Western blotting (Fig. 4, *E* and *F*, all *p* < 0.01). Then, we performed ChIP assays and found that knocking down *LINC00839* significantly reduced TAF15 enrichment at promoter region of *AOC1* gene (Fig. 4*G*, *p* < 0.01). In addition, TAF15 binding site located at promoter region of *AOC1* was predicted (Fig. 4*H*) and dual-luciferase report assays demonstrated that knockdown of *TAF15* significantly decreased the transcriptional activity of the *AOC1* wildtype promoter construct rather than the motif-deficient promoter construct (Fig. 4*I*).

Overall, our data indicate that the interaction of TAF15 and *AOC1* promoter region was dependent on the existence of *LINC00839* and *LINC00839* tethers TAF15 to *AOC1* promoter for its activation.

### AOC1 is involved in *LINC00839*/TAF15-mediated NPC progression

**Figure 5:**
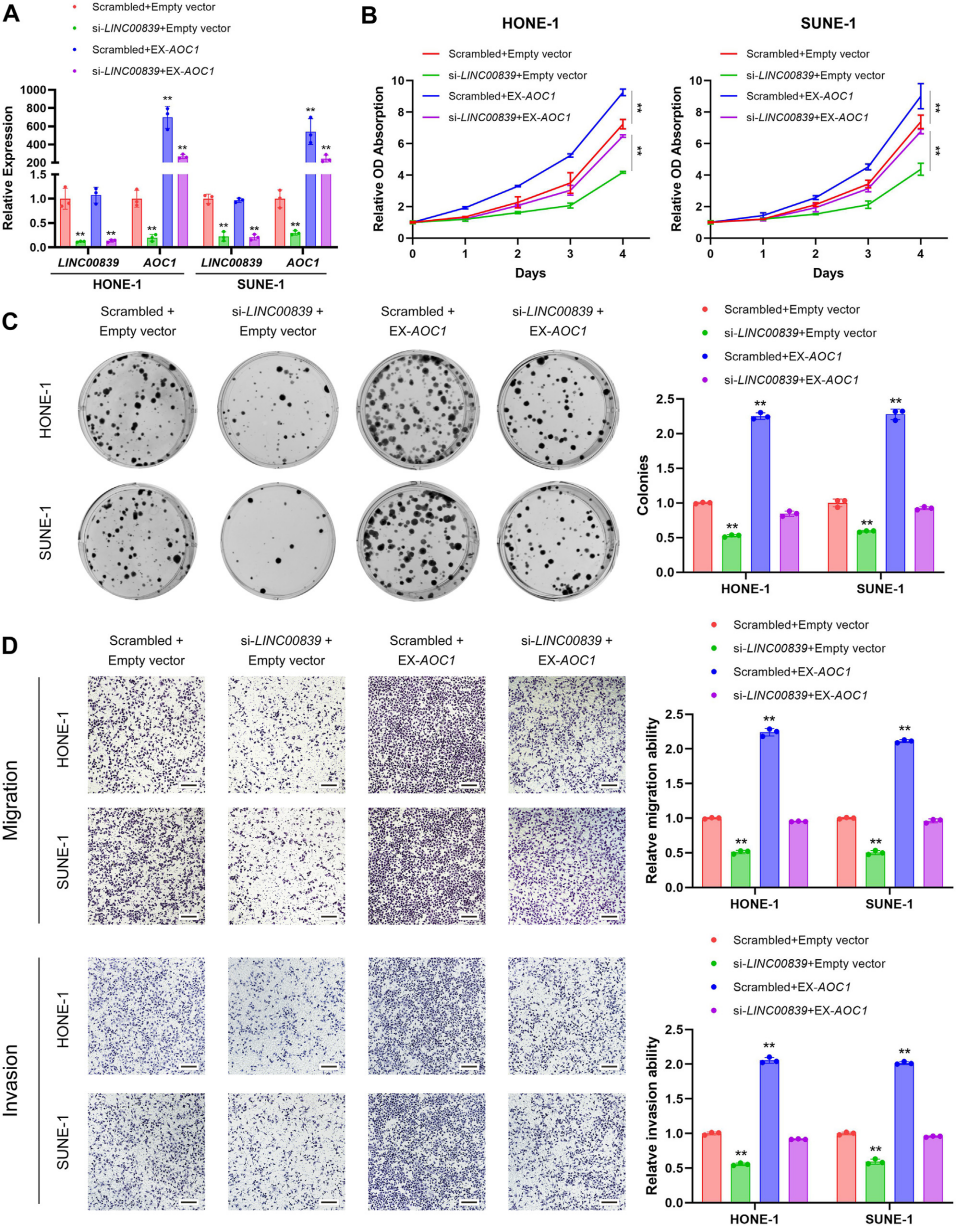
***AOC1* is involved in *LINC00839*/TAF15-mediated NPC progression.***A*, *LINC00839* and *AOC1* expression in HONE-1 and SUNE-1 cells cotransfected with si-*LINC00839* or the scrambled control, together with pUC57-*AOC1* or empty vector. *B*, CCK-8 assays in HONE-1 and SUNE-1 cells cotransfected with si-*LINC00839* or the scrambled control, together with pUC57-*AOC1* or empty vector. *C*, representative images (*left*) and quantification (*right*) of colonies formed in HONE-1 and SUNE-1 cells cotransfected with si-*LINC00839* or the scrambled control, together with pUC57-*AOC1* or empty vector. *D*, representative images (*left*) and quantification (*right*) of migratory or invasive cells in HONE-1 and SUNE-1 cells cotransfected with si-*LINC00839* or the scrambled control, together with pUC57-*AOC1* or empty vector. Scale bars for plot (*D*) represent 250 μm. Data are presented as the mean ± SD. ∗*p* < 0.05, ∗∗*p* < 0.01. The experiments were repeated at least three times independently. *AOC1*, amine oxidase copper-containing 1; CCK-8, Cell Counting Kit-8; NPC, nasopharyngeal carcinoma; TAF15, TATA-box binding protein associated factor.

**Supporting Figure S6:**
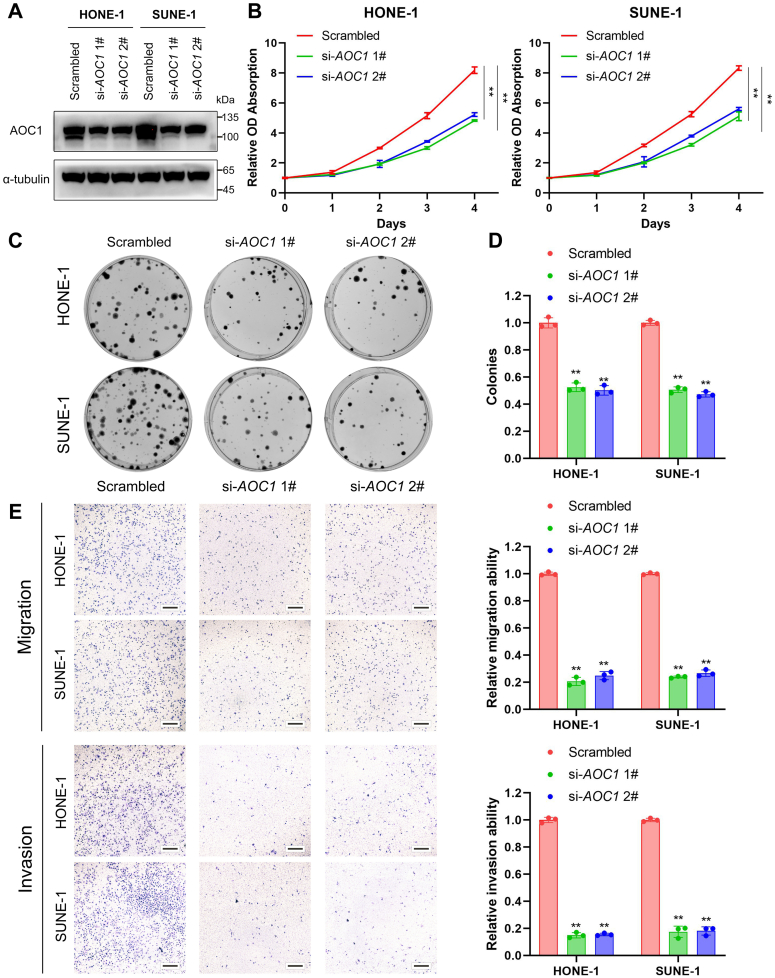
**AOC1 facilitates NPC proliferation, migration and invasion *in vitro*.***A*, AOC1 protein expression in HONE-1 and SUNE-1 cells transfected with si-*AOC1*s or the scrambled control. *B*, CCK8 assays in HONE-1 and SUNE-1 cells transfected with si-*AOC1*s or the scrambled control. *C* and *D*, Representative images (*C*) and quantification (*D*) of colonies formed in HONE-1 and SUNE-1 cells transfected with si-*AOC1*s or the scrambled control. *E*, Representative images (*left*) and quantification (*right*) of migratory (*upper*) or invasive (*lower*) cells in HONE-1 and SUNE-1 cells transfected with si-*AOC1*s or the scrambled control. Scale bars for plot (*E*): 250 μm. Data are presented as the mean ± SD. ∗*P* < 0.05, ∗∗*P* < 0.01. The experiments were repeated at least three times independently.

**Supporting Figure S7:**
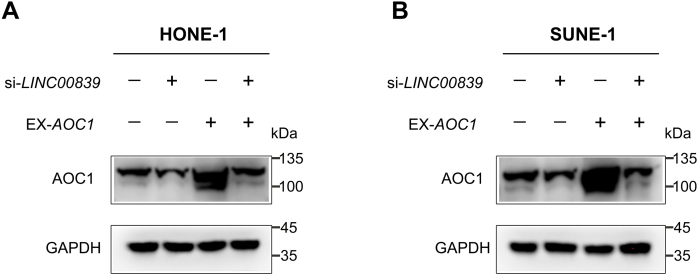
**Protein expression of AOC1.***A* and *B*, AOC1 protein expression in HONE-1 (*A*) and SUNE-1 (*B*) cells cotransfected with si-*LINC00839* or the scrambled control, together with pUC57-*AOC1* or empty vector.

To determine if AOC1 participates in NPC progression mediated by *LINC00839*/TAF15, we first examined the biological function of AOC1 in NPC. CCK-8, colony formation, and transwell assays manifested that knockdown of *AOC1* suppressed the proliferation, migration, and invasion of HONE-1 and SUNE-1 cells compared with the scrambled ones (Fig. S6). In addition, we transfected *AOC1* overexpressing plasmid into *LINC00839*-downregulated cells or scrambled ones, as expression level monitored by RT–qPCR and Western blotting (Figs. 5*A* and S7). Results illustrated that *AOC1* overexpression partially reversed the *LINC00839* knockdown–induced decline in NPC cell proliferation, migration, and invasion activities *in vitro* (Fig. 5, *B*–*D*).

### Silencing *LINC00839* inhibits NPC proliferation and metastasis *in vivo*

**Figure 6:**
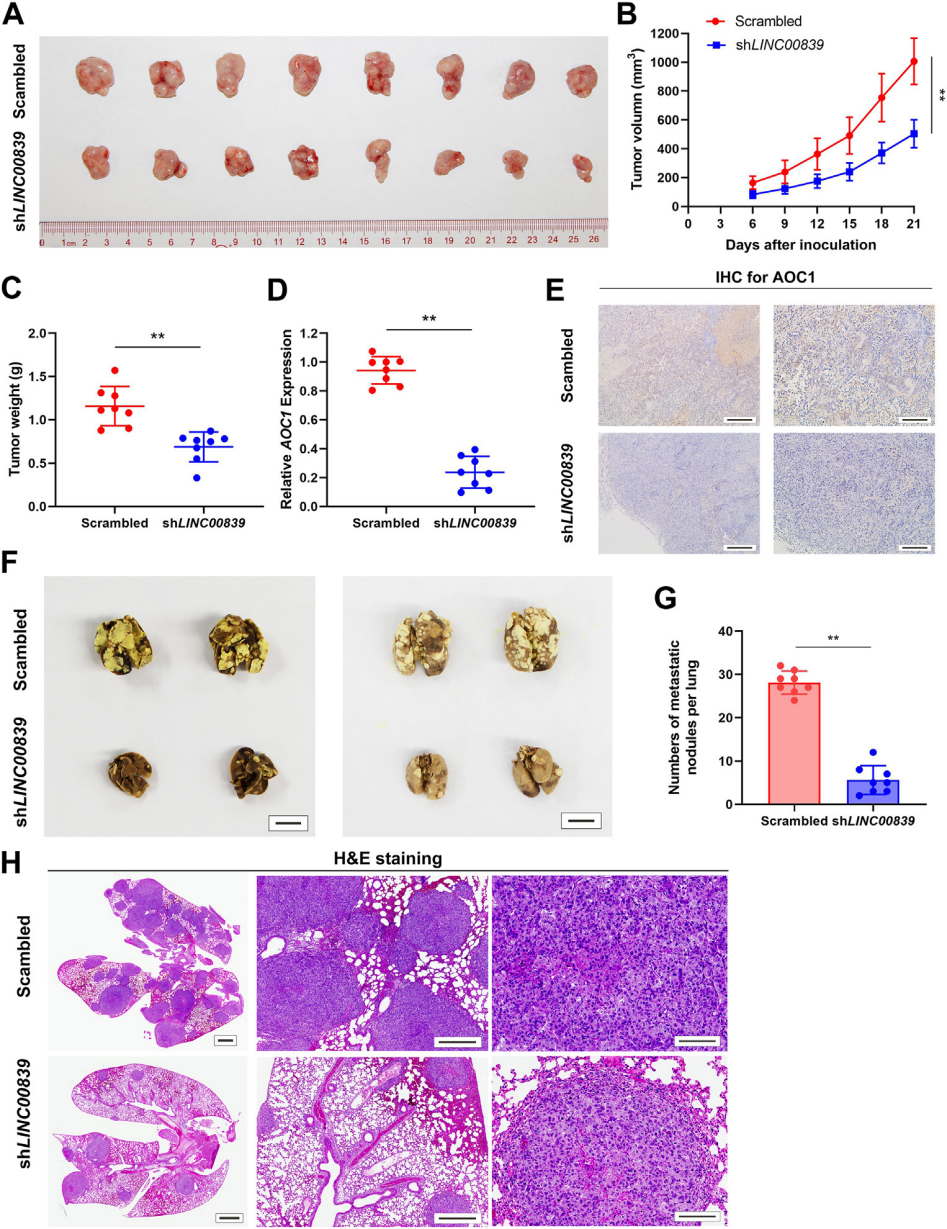
**Silencing *LINC00839* inhibits NPC proliferation and metastasis *in vivo*.***A*–*E*, SUNE-1-shNC or SUNE-1-sh*LINC00839* cells were inoculated into the axilla of nude mice (n = 8/group) to construct xenograft growth models. Representative image (*A*), tumor volume growth curves (*B*), and quantitative analysis of tumor weights (*C*) of xenograft tumors are presented. *LINC00839* and *AOC1* expression levels in the xenografts were detected using RT–qPCR (*D*) and IHC (*E*), respectively. Scale bars for plot (*E*) represent 250 μm (*left*) and 100 μm (*right*). *F*–*H*, SUNE-1-shNC or -sh*LINC00839* cells were injected through the tail vein of nude mice (n = 8/group) to construct metastatic colonization models. Representative image (*F*), statistical analysis of number of lung metastatic nodules (*G*), and H&E staining (*H*) are presented. Scale bars for plot (*F*) represent 1 cm. Scale bars for plot (*H*) represent 2 mm (*left*), 250 μm (*middle*), and 50 μm (*right*). Data are presented as the mean ± SD. ∗*p* < 0.05, ∗∗*p* < 0.01. The experiments were repeated at least three times independently. *AOC1*, amine oxidase copper-containing 1; IHC, immunohistochemistry; NPC, nasopharyngeal carcinoma; qPCR, quantitative PCR.

To further clarify the oncogenic effect of *LINC00839* on NPC tumor *in vivo*, we established stable *LINC00839* knockdown SUNE-1 cells with shRNAs, which were then inoculated into nude mice to construct xenograft growth models. Notably, xenografts of *LINC00839* knockdown group exhibited significantly reduced tumor growth rate, tumor volume, and tumor weight relative to scrambled control group (Fig. 6, *A*–*C*, all *p* < 0.01). Further analyses validated that the knockdown of *LINC00839* resulted in a reduction in *AOC1* levels (Fig. 6, *D* and *E*, *p* < 0.01). Moreover, we constructed lung metastatic colonization models *via* injecting cells through the tail vein of nude mice. The number of metastatic nodules on lung surface in *LINC00839* silencing group was markedly fewer than that in control group (Fig. 6, *F* and *G*, *p* < 0.01). In addition, HE staining showed that silencing *LINC00839* contributed to a less aggressive phenotype and a significantly lower metastasis ratio of lung nodules (Fig. 6*H*). Taken together, these results indicate that *LINC00839* acts as an oncogene and promotes NPC proliferation and metastasis *in vivo*.

### VIRMA and IGF2BP1 stabilize *LINC00839* in an m6A-dependent manner

**Figure 7:**
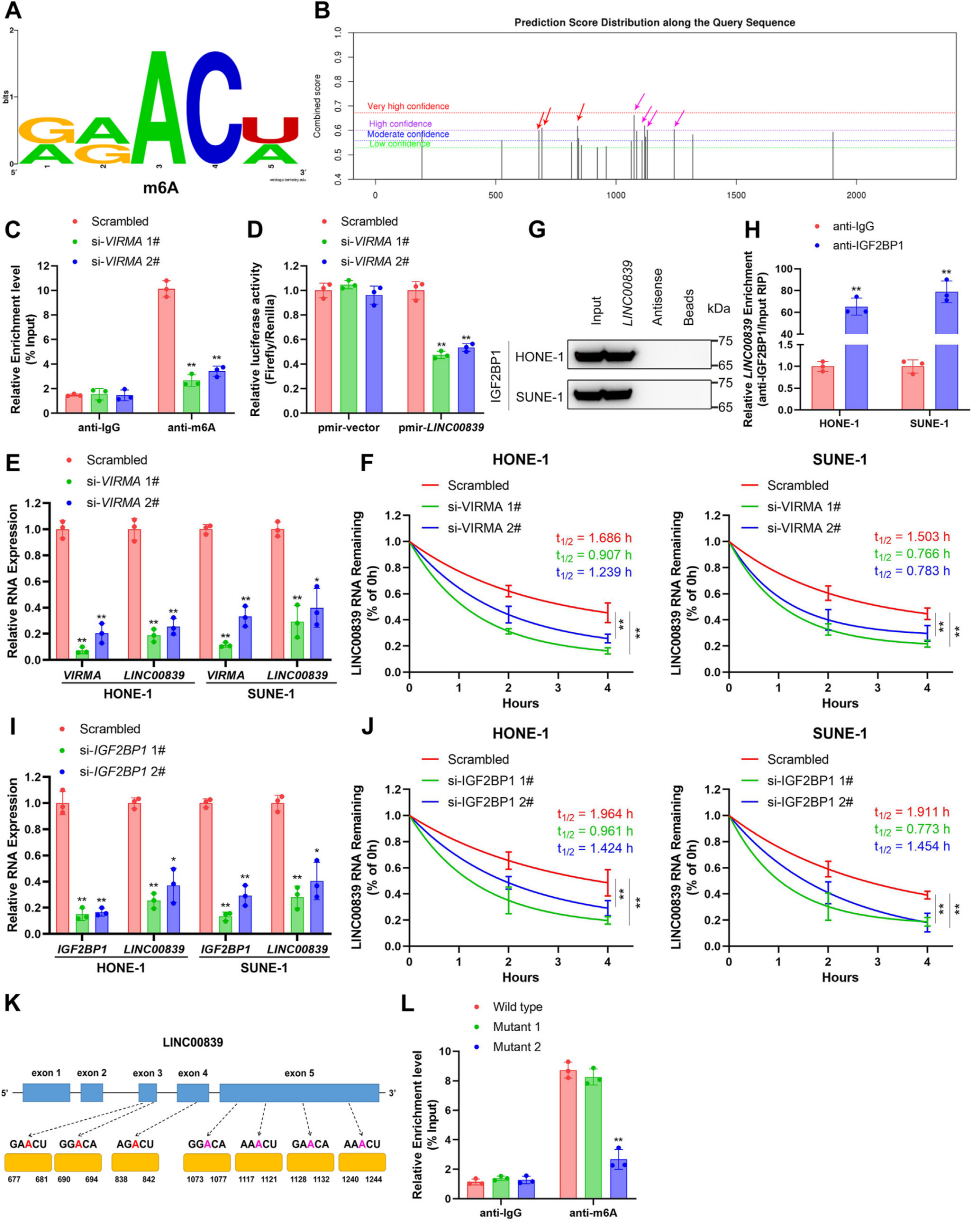
***VIRMA* and IGF2BP1 stabilized *LINC00839* in an m6A modification–dependent manner.***A*, predicted m6A motifs with high confidence in *LINC00839* transcripts. *B*, the enriched and specific m6A peak distribution of *LINC00839* predicted by SRAMP. *Arrows* indicate the m6A enrichment peaks in exon 3‒4 (*red*) and exon 5 (*purple*) of *LINC00839* transcripts. *C*, m6A enrichment in *LINC00839* transcripts (500∼1000 nt) in control and *VIRMA*-knockdown cells using MeRIP–qPCR. *D*, relative luciferase activity in HONE-1 cells cotransfected with pmirGLO-*LINC00839* or pmirGLO-vector (luciferase reporter) and si-*VIRMA*s or the scramble control. *E*, interaction of *LINC00839* and IGF2BP1 was confirmed by RNA pull-down and Western blotting analysis. *F*, representative images (*left*) and quantification (*right*) of colonies formed in HONE-1 and SUNE-1 cells transfected with pcDNA3.1(−)-*LINC00839* or empty vector. *G*, *VIRMA* and *LINC00839* expression in HONE-1 and SUNE-1 cells transfected with si-*VIRMA*s or the scrambled control. *H*, *LINC00839* RNA stability in control and *VIRMA*-silenced cells. *LINC00839* expression levels were monitored using RT–qPCR at various time points after actinomycin D (10 μg/ml) treatment. *I*, *IGF2BP1* and *LINC00839* expression in HONE-1 and SUNE-1 cells transfected with si-*IGF2BP1*s or the scrambled control. *J*, *LINC00839* RNA stability in control and *IGF2BP1*-silenced cells. *LINC00839* expression levels were monitored using RT–qPCR at various time points after actinomycin D (10 μg/ml) treatment. *K*, diagram showing the position of m6A motifs with a high combined score within *LINC00839* transcripts. *L*, MeRIP–qPCR assays to analyze the m6A modification levels of *LINC00839* in HONE-1 cells transfected with *LINC00839* wildtype and its mutants. Mutant 1, predicted m6A motifs in exon 3‒4 were substituted by cytosine (A‒C mutant); mutant 2, predicted m6A motifs in exon 5 were substituted by cytosine (A‒C mutant). Data are presented as the mean ± SD. ∗*p* < 0.05, ∗∗*p* < 0.01. The experiments were repeated at least three times independently. *IGF2BP1*, insulin-like growth factor 2 mRNA-binding protein 1A; m6A, N6-methyladenosine; MeRIP, methylated RNA immunoprecipitation; qPCR, quantitative PCR; SRAMP, Sequence-based RNA Adenosine Methylation Site Predictor; *VIRMA*, vir-like m6A methyltransferase-associated.

Recent studies have elucidated that the m6A modification is widely distributed in RNA and exerts a modulatory role in the expression, localization, and stabilization of lncRNA (36, 37). Therefore, we studied whether *LINC00839* upregulation in NPC was associated with m6A modification. We first predicted the m6A sites of *LINC00839* using an online bioinformatics method SRAMP (http://www.cuilab.cn/). We found enriched m6A peaks along *LINC00839* sequence. Besides, seven m6A sites (RRACH motifs) with high confidence scores were detected at the region of 679- to 1242-nt (exon 3‒5) (Fig. 7, *A* and *B*).

**Supporting Figure S8:**
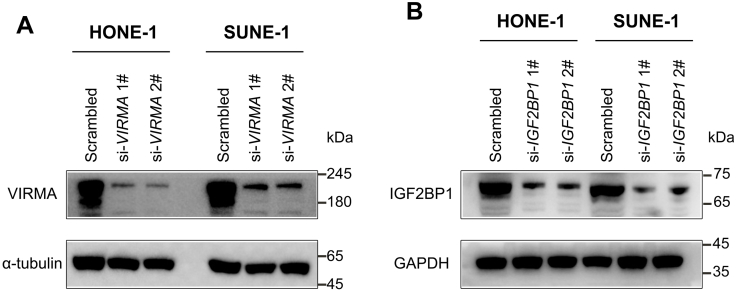
**Protein expression of VIRMA and IGF2BP1.***A*, VIRMA protein expression in HONE-1 and SUNE-1 cells transfected with si-*VIRMA*s or the scrambled control. *B*, IGF2BP1 protein expression in HONE-1 and SUNE-1 cells transfected with si-*IGF2BP1*s or the scrambled control.

VIRMA was previously claimed as a component of m6A methyltransferase complex, which was critical for m6A catalytic process (18). We conducted methylated RNA immunoprecipitation (MeRIP) using m6A antibody in HONE-1 cells with or without *VIRMA* knockdown and found that m6A modification levels of *LINC00839* were markedly reduced in *VIRMA*-downregulated cells (Fig. 7*C*, *p* < 0.01). In accordance with these results, dual-luciferase reporter assays demonstrated that downregulation of *VIRMA* substantially inhibited luciferase activity of *LINC00839* reporter construct (Fig. 7*D*, *p* < 0.01). We further investigated whether the m6A modifications affect expression of *LINC00839*. Consistent with previous results, silencing *VIRMA* significantly suppressed *LINC00839* expression (Figs. 7*E* and S8, *p* < 0.01). Moreover, knocking down *VIRMA* decreased the half-life of *LINC00839* in HONE-1 and SUNE-1 cells treated with actinomycin D (Fig. 7*F*, *p* < 0.01). The half-life of *LINC00839* was approximately 1.5 to 2.0 h in control cells, whereas silencing *VIRMA* accelerated *LINC00839* decay by about 25 to 50% (0.8‒1.5 h).

MS analysis identified IGF2BP1 among *LINC00839* pull-down proteins, which belongs to the IGF2BP family known as m6A readers, recognizes m6A sites, and guards m6A-modified RNAs from decay (18). Interaction between *LINC00839* and IGF2BP1 was confirmed by RNA pull-down and Western blotting (Fig. 7*G*). Moreover, RIP assays manifested that IGF2BP1 significantly enriched *LINC00839* (Fig. 7*H*, *p* < 0.01). Furthermore, silencing *IGF2BP1* conferred apparently decreased expression and slower degradation rate of *LINC00839* compared with the control cell (Figs. 7, *I* and *J* and S8, all *p* < 0.01).

**Supporting Figure S9:**
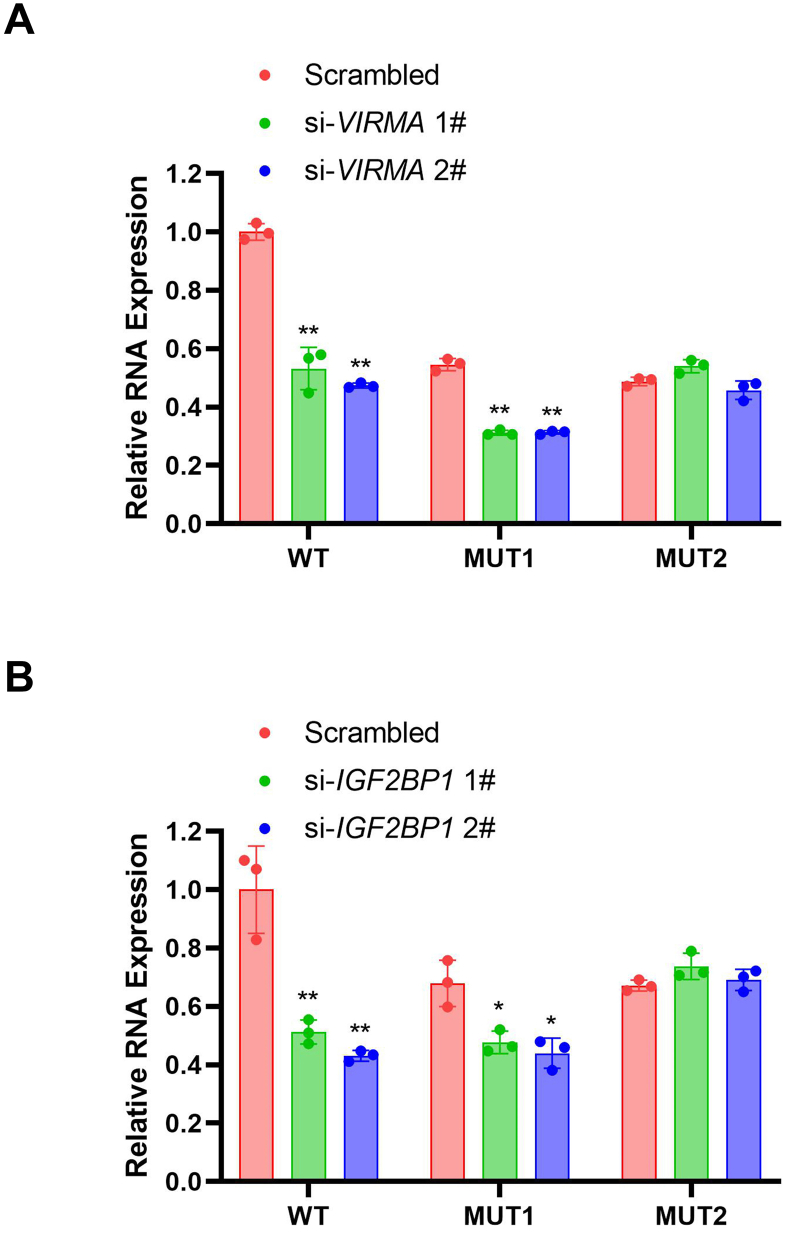
**Specific m6A sites related to the modulation of *LINC00839* expression.***A*–*B*, Knocking down *VIRMA* (*A*) or *IGF2BP1* (*B*) in the wild-type and mutant constructs overexpressing HONE-1 cells reduced the expression of wild-type *LINC00839* and Mutant 2, but did not influence the expression of Mutant 1.

**Supporting Figure S10:**
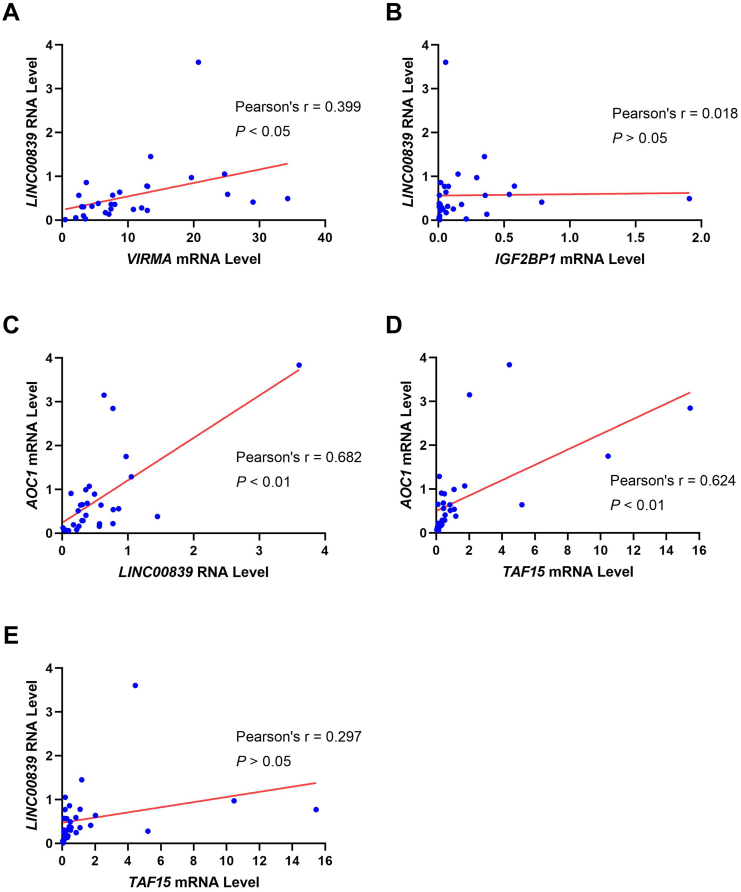
**Pearson correlation analyses of 30 NPC clinical specimens among VIRMA/IGF2BP1–*LINC00839*–TAF15–AOC1 axis.***A*–*E*, Pearson correlation analyses of *VIRMA* and *LINC00839* (*A*); *IGF2BP1* and *LINC00839* (*B*); *LINC00839* and *AOC1* (*C*); *TAF15* and *AOC1* (*D*); *TAF15* and *LINC00839* (*E*); expression in 30 NPC clinical specimens.

To investigate specific m6A motifs that m6A modification is dependent on, we constructed wildtype and two mutant *LINC00839* vectors (mutant-1: exon 3‒4; mutant-2: exon 5), in which the adenine residues in predicted m6A motifs of *LINC00839* were substituted by cytosine (A‒C mutant) (Fig. 7*K*). MeRIP–qPCR presented that m6A modifications on *LINC00839* mutant-2 transcript were obviously declined with respect to the wildtype transcript, whereas m6A levels on mutant-1 transcript were comparable to the wildtype one (Fig. 7*L*, *p* < 0.01). Besides, we knocked down *VIRMA* or *IGF2BP1* in the wildtype and mutant constructs overexpressing HONE-1 cells and found that either silencing *VIRMA* or *IGF2BP1* reduced the expression of wildtype *LINC00839* and mutant 2, which have the essential m6A residues, but not mutant 1 (Fig. S9). The results illustrate that the m6A motifs located within exon 5 (1075–1242 nt) on *LINC00839* are predominantly responsible for its regulation. Moreover, we detect the expression levels of *VIRMA* and *IGF2BP1* in the 30 NPC clinical specimens. The results to some extent verified correlations between m6A modulations and expression of *LINC00839* (Fig. S10).

In summary, VIRMA (m6A writer) and IGF2BP1 (m6A reader) increase *LINC00839* stability and thereby facilitating expression *via* an m6A-dependent manner.

## Discussion

To elucidate the underlying molecular mechanisms of carcinogenesis and progression, quantities of studies concerning genetic aberrations and epigenetic dysregulations have been conducted (38, 39). Among them, lncRNAs have attracted increasing interests because of their diverse roles proposed in malignant transformation (11). Nevertheless, the clinical prognostic value and biological function of lncRNAs still remain ambiguous in NPC. In this study, we reanalyzed the lncRNA expression profiles of NPC tissues and recognized an m6A-modified lncRNA, *LINC00839*, which was markedly elevated in NPC cells and tissues. *LINC00839* is a long intergenic noncoding RNA situated on10q11.21. Several studies described that *LINC00839* was significantly elevated and could promote tumor progression in breast cancer, hepatocellular carcinoma, osteosarcoma, and neuroblastoma (26, 27, 28, 29). Here, *in vitro* and *in vivo* experiments stipulated that *LINC00839* promoted NPC growth and metastasis. Multivariate survival analyses suggested that high *LINC00839* expression was an adverse prognostic factor for NPC patients independent of general clinical features. Given these observations, we considered *LINC00839* as a critical oncogenic modulator in NPC initiation and development.

Gene regulation is modulated by lncRNAs at multiple levels by interacting directly with DNA, RNA, or protein (10). LncRNAs are recognized as crucial mediators functioning in numerous biological processes, such as chromatin remodeling (40), transcription modulation in *cis* or in *trans* (9), and formation of organelles or nuclear condensates (41, 42), and so on. Previously, a number of researches reported that lncRNAs could interact with and regulate the behavior of transcription-associated protein and regulate gene expression in *trans*. For example, *HOTAIR* directly interacted with polycomb repressive complex 2 and functioned as a scaffold coordinating the occupancy of polycomb repressive complex 2 on the HOXD locus, which was responsive for histone H3 lysine-27 trimethylation. Through the recruitment and histone methylation, *HOTAIR* suppressed downstream genes transcription in *trans* (12). *LincRNA-EPS* was found to directly interact with the promoters of target genes, recruit heterogeneous nuclear ribonucleoproteinribonucleoprotein L to gene promoter and play an important role in subsequent transcriptional repression (43). In addition, lncRNA *SLERT*, highly enriched in nucleus, was reported to interact with DDX21 and activate Pol I transcription. Further analysis found that DDX21 formed ring-like structures around Pol I and repressed transcription, whereas the interaction of *SLERT* and DDX21 antagonized this regulation (44). Recently, it has been reported that *lnc-CTHCC* binds to transcription factor heterogeneous nuclear RNP K and activates *YAP1* transcription and promotes progression of hepatocellular carcinoma (45). With regard to lncRNAs in NPC, LINC01503 was illustrated to stimulate NPC growth and development by splicing factor proline–glutamine rich–mediated transcription activation and upregulated expression of *FOSL1* (46). In this study, we found that *LINC00839* directly interacted with a transcription factor, TAF15, and tethered TAF15 to promoter region of *AOC1*, which subsequently facilitated transcription activities and enhanced the expression of *AOC1*.

TAF15 is a conserved RNA-binding protein belonging to the multifunctional TET protein family. It is composed of an N-terminal LC domain, RGG domains, a zinc finger domain, and an RNA recognition motif, among which RRM domain was distinguished to contribute to recognition of RNA (30). With capacity of DNA and RNA binding, TAF15 has been described to affect RNA transcription by directly binding of RNA Pol II or other transcription components (31, 32). Moreover, TAF15 has been reported to be involved in cell spreading and cell adhesion and implemented in cellular process regarding malignancy (47, 48). It has also been reported to interact with lncRNAs and serve as an oncogene in various cancers (49, 50, 51). For instance, LINC01048 increases the binding of TAF15 to *YAP1* promoter and gives rise to sustaining activation of Hippo pathway and hence promotes cell proliferation in cutaneous squamous cell carcinoma (49). Our results demonstrated that TAF15 could promote NPC growth and metastasis. Interaction and colocalization in nucleus of *LINC00839* and TAF15 was proved in NPC cells. We also discerned the specific region of *LINC00839* (1059- to 1552-nt) responsible for binding to TAF15. Besides, we found that changes of *LINC00839* expression had no effect on the expression of TAF15. Since KEGG analysis indicated that *LINC00839* might exert influence on transcriptional misregulation in cancer, we performed ChIP assays and detected that *LINC00839* increased the occupancy of TAF15 to promoter region of *AOC1*. Previous studies demonstrated that lncRNA could promote FET proteins to oligomerize and then activate the direct binding of FET protein and RNA Pol II (52, 53, 54). Besides, lncRNA could also act as scaffold for interaction of FET proteins and other transcription factors, which might activate or silence transcription (55, 56). Recently, a series of studies have focused on the phase separation process and transcriptional activity of FET proteins (32, 57). However, in this study, we did not show further mechanisms because of the technical limitations. We believe that further explorations on this field are meaningful and needful.

AOC1, a homodimeric glycoprotein, is one of the amine oxidases that catalyze the deamination of putrescine, histamine, and related compounds (33). Amine oxidases were previously reported to be related to tumor growth and progression. In breast cancer, positive expression of AOC3 was associated with higher pathological grade and Ki-67 expression (58). AOC1-induced changes in polyamine homeostasis affected embryonic kidney morphogenesis regulated by Wilms tumor protein (59). In addition, AOC1 was also found promoting cancer progression in gastric and colorectal cancer by activating AKT pathway and epithelial–mesenchymal transition process (34, 35). We discovered that AOC1 promotes NPC proliferation and metastasis. Either silencing *LINC00839* or *TAF15* decreased the expression of AOC1. Besides, TAF15 and *LINC00839* were proved to be vital for *AOC1* transcription, since knocking down *TAF15* significantly inhibited *AOC1* transcription activities and *LINC00839* was responsible for recruitment of TAF15 to *AOC1* promoter. Ectopic expression of *AOC1* partially rescued the inhibitory effect of downregulation of *LINC00839* in NPC. Overall, we propose that *LINC00839* promotes NPC progression by tethering TAF15 to *AOC1* promoter and increases AOC1 expression.

Recently, m6A, a predominant RNA epigenetic modification, has been found participating in various biological processes in carcinogenesis and development. Particularly, core molecular mechanisms of this reversible process consist of m6A methylation, demethylation, and recognition of specific m6A motif. Moreover, relevant m6A profiling studies identified noncoding RNAs as targets of this modification (36, 37). For example, lncRNA *NEAT1-1* promotes bone metastasis of prostate cancer *via* modulating CYCLINL/CDK19 complex, reliant on m6A-modified sites (60). A recent study showed that WTAP promoted proliferation and metastasis of NPC *via* mediating *DIAPH1-AS1* in an m6A-dependent manner (24). In this study, related data illustrated that VIRMA/IGF2BP1-mediated m6A enrichment accounted for the high expression and RNA stability of *LINC00839*. We confirmed the direct interaction between IGF2BP1 and *LINC00839*. Either knocking down *VIRMA* or *IGF2BP1* significantly reduced expression and stimulated degradation of *LINC00839*. Further analysis also implied that m6A modification (consensus RRACH sequence) within exon 5 of *LINC00839* might be dominant for this regulating process.

**Figure 8:**
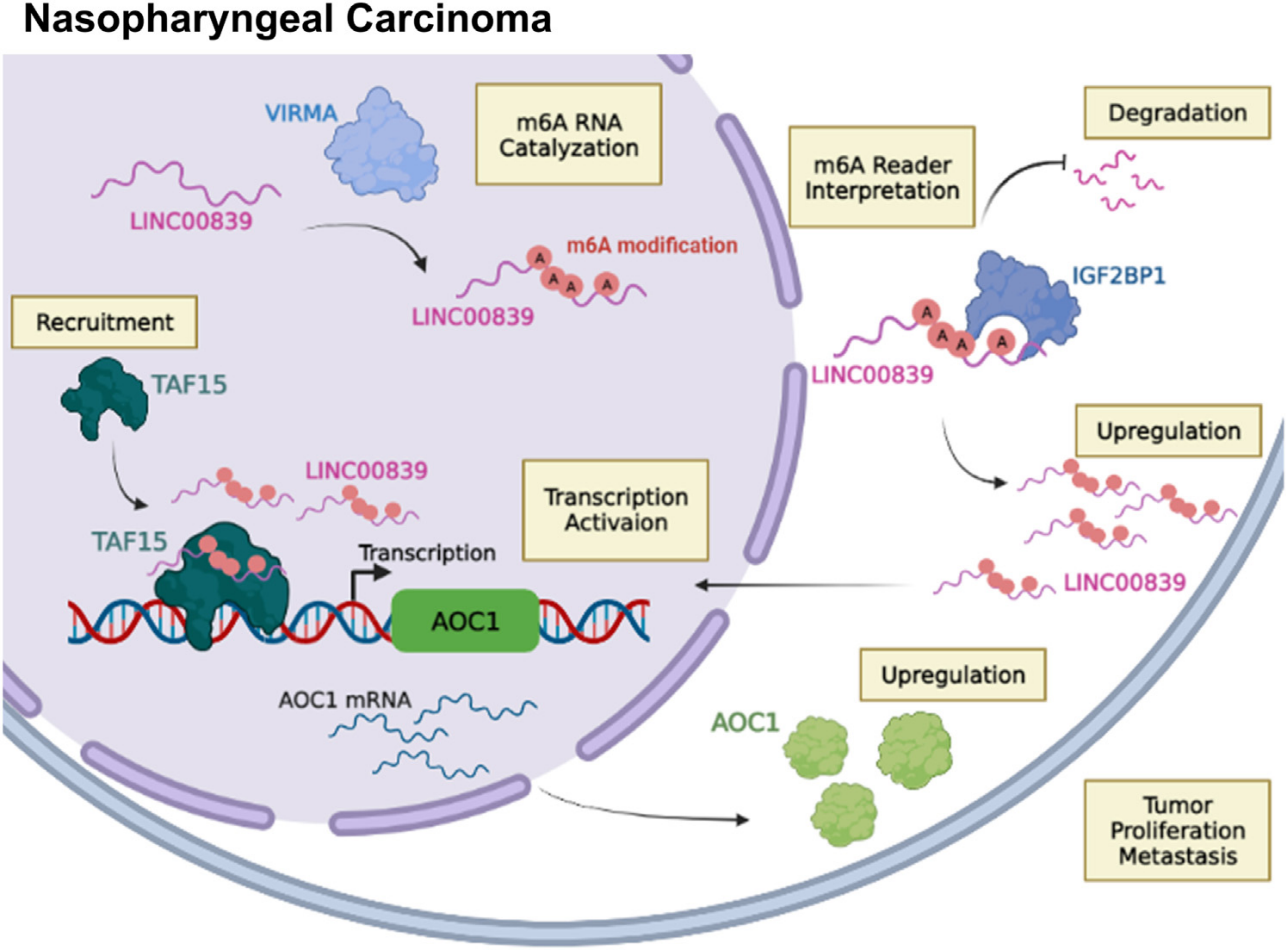
**A graphic exemplification of VIRMA/IGF2BP1–*LINC00839*–TAF15–AOC1 axis.***LINC00839* promoting NPC progression by recruiting transcription factor TAF15 to the promoter region of *AOC1* and boosting transcription and expression of *AOC1*. VIRMA and IGF2BP1 contribute to the RNA stability and upregulation of *LINC00839* in an m6A-dependent manner. AOC1, amine oxidase copper-containing 1; IGF2BP1, insulin-like growth factor 2 mRNA-binding protein 1A; NPC, nasopharyngeal carcinoma; TAF15, TATA-box binding protein associated factor; VIRMA, vir-like m6A methyltransferase-associated.

In conclusion, our results uncovered a molecular mechanism model whereby *LINC00839* dictates the transcriptional status and affects expression of *AOC1* by recruiting TAF15, thereby promoting NPC growth and metastasis ultimately. In addition, VIRMA/IGF2BP1-modulated m6A modifications maintain *LINC00839* stability and high expression in NPC (Fig. 8). Accordingly, *LINC00839* might potentially serve as a prognostic biomarker or a therapeutic target for individualized treatment of NPC.

## Experimental procedures

### Clinical specimens

This study was approved by the Institutional Review Board of Sun Yat-Sen University Cancer Center (GZR2022-330), and written informed consent was obtained from all the patients. Totally 30 freshly frozen NPC and 10 normal nasopharyngeal epithelial tissues were obtained from the Pathology Department of Sun Yat-sen University Cancer Center. All formalin-fixed and paraffin-embedded tissues of human NPC biopsy (n = 214) were obtained from patients with detailed clinical characteristics and long-term follow-up data from January 2004 to December 2013. The median follow-up time of all patients was 70.9 months (range = 2.6–115.9 months).

### Cell culture

All cell lines had been authenticated and were generously provided by Dr M. Zeng (Sun Yat-sen University Cancer Center). Two human immortalized nasopharyngeal epithelial cell lines (NP69 and N2Tert) were cultured in keratinocyte serum-free medium (Invitrogen) supplemented with bovine pituitary extract (BD Biosciences). All human NPC cell lines (HONE-1, SUNE-1, C666, HNE-1, 5-8F, 6-10B, S18, S26, CNE-1, CNE-2, and HK-1) were cultured under recommended conditions: RPMI1640 medium (Invitrogen) or Dulbecco's modified Eagle's medium (Invitrogen) supplemented with 10% fetal bovine serum (Invitrogen). Among them, SUNE-1 and HONE-1 cell lines have been considered authentic human NPC cell lines, which were derived from Epstein–Barr virus–positive and poorly differentiated NPC primary cultures (61, 62, 63).

All cell lines were maintained in an incubator with a humidified atmosphere of 95% air and 5% CO_2_ at 37 °C. Cells were passaged every 2 to 3 days and kept in culture for the duration of the experiments. Cells were stored in liquid nitrogen using CELLSAVING (New Cell & Molecular Biotech). Cell line experiments were performed within 1 to 2 months of their resuscitation.

### RNA extraction, RT, and real-time RT–qPCR

Total RNA was extracted from tissue samples and cell lines using TRIzol reagent (Invitrogen). Complementary DNA (cDNA) was generated using HiScript III RT SuperMix (Vazyme). A mix of primer pairs, double-distilled water, and 2× SYBR Green qPCR SuperMix-UDG reagents (Invitrogen) was added to the cDNA, and amplified copies were quantified by CFX96 Touch real-time system (Bio-Rad) or LightCycler 480 System (384 wells; Roche) according to the manufacturers’ instructions. The sequences of primers used for RT–qPCR are listed in Table S3. The cycle number (CQ) values were normalized against that of *GAPDH.* All reactions were conducted in triplicate.

### Western blotting

Cell lysates were extracted using radioimmunoprecipitation assay buffer (Millipore) containing protease and phosphatase inhibitors (Thermo Fisher Scientific). Protein samples were mixed with 5× loading buffer, boiled for 20 min at 95 °C, run on 4 to 20% SDS-polyacrylamide gel (Genescript), transferred to polyvinylidene fluoride membranes (Millipore), blocked with 5% skimmed milk, and incubated overnight at 4 °C with the corresponding primary antibody. Membranes were washed three times for 10 min each with 1× Tris-buffered saline with Tween-20 buffer and then incubated for 1 h at room temperature with secondary antibodies (Abcam/Proteintech). Membranes were washed again three times for 10 min with 1× Tris-buffered saline with Tween-20 before revealing them with a chemiluminescence detection kit (Yeason Biotechnology) and analyzing them on a ChemiDoc Touch Imaging System (Bio-Rad). The antibodies used in this study: anti-TAF15 (1:10,000 dilution; Abcam), anti-AOC1 (1:1000 dilution; Abcam), anti-VIRMA (1:1000 dilution; Abcam), anti-IGF2BP1 (1:1000 dilution; Abcam), anti-α-tubulin (1:5000 dilution; Abcam), and anti-GAPDH (1:5000 dilution; Abcam).

### Cell transfection and lentiviral infection

Duplex RNAi oligonucleotides targeting human *LINC00839*, *TAF15*, *VIRMA*, *IGF2BP1*, and *AOC1* were synthesized by RiboBio or GenePharma, of which sequences were shown in Table S4. A scrambled duplex RNA oligonucleotide was used as an RNA negative control. For gene overexpression, cDNAs comprising the open reading frames of human *AOC1* and *TAF15* (full-length and truncated variants) with sequences encoding an N-terminal FLAG tag were purchased from Tsingke Biotechnology. The full-length sense and antisense sequences of *LINC00839* were synthesized by Genscript and cloned into the pcDNA3.1(−) and pcDNA3.1(+) vectors to generate overexpression plasmids, respectively. The truncated variants of *LINC00839* were purchased from Tsingke Biotechnology (pcDNA3.1(−) vector). The m6A methylated site mutations (wildtype, mutant 1, and mutant 2) of *LINC00839* were synthesized by Genscript and cloned into the pmir-GLO vectors to generate dual-luciferase vectors. Corresponding empty vectors were used as the negative control. Cells were transfected with the siRNA using Lipofectamine RNAiMAX (Invitrogen) or with plasmids using Lipofectamine 3000 (Invitrogen) according to the manufacturers’ instructions. In addition, the shRNA plasmids against *LINC00839* were synthesized according to the sequences shown in Table S5, and sh*LINC00839*-2# was inserted into vector pLKO.1. Next, psPAX2 packaging plasmid (Addgene) and the pCMV-VSV-G envelope plasmid (Addgene) were mixed with pLKO.1-sh*LINC00839*-2# for transfection of human embryonic kidney 293T cells using polyethyleneimine (Polysciences). Supernatants were collected after 24 h and used to infect SUNE-1. Following the infection, target cells were selected with 1 μg/ml puromycin (Thermo Fisher Scientific) for 7 days and validated using RT–qPCR.

### RNA-Seq and bioinformatic analysis

Samples for RNA-Seq were directly extracted from SUNE-1 cells transfected with siRNA against *LINC00839*. RNA-Seq was conducted by Lc-bio Technologies. Differentially expressed genes (|log_2_ fold change| >1 and *p* < 0.05) were identified and subjected to Gene Ontology (Fig. S1) and KEGG pathway analyses using the DAVID software (https://david.ncifcrf.gov/).

### Cell proliferation and colony-formation assays

For CCK-8 assay, transfected cells were seeded in 96-well plates at a density of 1000 cells per well, and cell viability was evaluated every 24 h for 5 days using a CCK-8 assay according to the manufacturers’ instructions (Dojindo). For the colony-formation assay, transfected cells were seeded in 6-well plates with density of 400 cells per well and cultured for approximately 10 days. The cells were then fixed with methanol, stained with hematoxylin, and photographed. Results were analyzed using ImageJ software (free open source developed by National Institutes of Health).

### Transwell migration and invasion assay

For Transwell assays, transfected cells (5 × 10^4^ for migration; 1 × 10^5^ for invasion) were suspended in 200 ml of serum-free medium and were seeded in the upper chamber of Transwell chambers (Corning) coated without or with matrigel (BD Biosciences). Medium containing with 10% fetal bovine serum was added to the lower chamber. After incubation (12 h for migration; 24 h for invasion), the cells located on the lower surface of the membrane were fixed with methanol, stained with hematoxylin, and photographed with inverted microscope.

### RNA pull-down assay and MS analysis

RNA pull-down assays were performed using a Pierce Magnetic RNA-Protein Pull-Down kit (Thermo Fisher Scientific) according to the manufacturer’s instructions. *In vitro* transcription and biotin RNA labeling of full-length sense, antisense, and variants of *LINC00839* was conducted with Ribo RNAmax-T7Biotin-labeling transcription kit (RiboBio). Biotinylated RNA was incubated with magnetic beads and then incubated with cell lysates at 4 °C overnight. The purified proteins were analyzed by MS and Western blotting. The proteins discovered by MS are listed in Tables S6 and S7.

### RIP

RIP assay was performed using a Magna RIP RNA-Binding Protein Immunoprecipitation kit (Millipore) according to the manufacturers’ instructions. Briefly, immunoglobulin G antibody (Millipore), anti-TAF15 (Cell Signaling Technology), or anti-IGF2BP1 (Abcam) was conjugated to magnetic beads and incubated with the corresponding cell lysates at 4 °C overnight. The coprecipitated RNAs were purified by resuspending beads in TRIzol regent and finally subjected to RT and RT–qPCR.

### Subcellular fractionation and FISH

Nuclear and cytoplasmic RNA was separated with the NE-PER Nuclear and Cytoplasmic Extraction Reagents (Invitrogen) and then analyzed by RT–qPCR. For FISH assays, cells were first grown for 24 h in 24-well plates with glass cover slips. After immobilization and permeabilization, cells were hybridized with 20 μM Cy3-labeled *LINC00839* probe (RiboBio), and 6-diamidino-2-phenylindole was used to stain nuclei. Images were observed with an LSM 980 confocal laser-scanning microscope (ZEISS).

### ChIP and ChIP sequencing

The ChIP assay was performed using a Pierce Magnetic ChIP Kit (Thermo Fisher Scientific). Cells were fixed with 1% formaldehyde and quenched with glycine, respectively. Nuclei were harvested and sonicated to generate DNA fragments of ∼200 bp. The sonicated chromatin was immunoprecipitated with anti-TAF15 (Cell Signaling Technology) antibody. Immunoprecipitated DNA was purified with spin columns and then analyzed by RT–qPCR. The primer sequences are listed in Table S8.

ChIP-Seq was conducted by Lc-bio Technologies. The high-throughput DNA sequencing libraries were prepared using VAHTS Universal DNA Library Prep Kit for Illumina V3 (catalog no.: ND607; Vazyme). The library products corresponding to 200 to 500 bps were enriched, quantified, and finally sequenced on Novaseq 6000 sequencer (Illumina) with PE150 model. Raw sequencing data were filtered by Trimmomatic (version 0.36), and clean data were mapped to reference genome using STAR (version 2.5.3a). Read distribution analysis, peak calling, and peak annotation/peak distribution analysis were performed by RSeQC (version 2.6), MACS2 (version 2.1.1), and bedtools (version 2.25.0), respectively. The Homer (version 4.10) was used for motif analysis. All packages were open sources from python software.

### Luciferase reporter assays

For *AOC1* wildtype and mutant promoter, we first predicted the potential binding sites using the Homer software (version 4.10) and identified totally seven motifs located at promoter region of *AOC1*, which might be accounting for TAF15 binding. Next, we constructed *AOC1* wildtype promoter (−2000 to 0 nt) and the corresponding motif-deficient promoter region onto luciferase reporter vectors (pGL3-Basic). After screening by dual-luciferase report assays, we recognized two binding sites located on promoter region essential for TAF15 and *AOC1* interaction (Fig. 5*H*). The promoter region and binding-site deleted mutations (wildtype, mutant) of *AOC1* were synthesized by Tsingke Biotechnology and cloned into the pGL3-Basic vectors.

For m6A-relevant luciferase report vector, we first predicted the m6A sites of *LINC00839* using an online bioinformatics method SRAMP (http://www.cuilab.cn/) and found enriched m6A peaks along *LINC00839* sequence. Seven m6A sites (RRACH motifs) with high confidence scores were detected at the region of 679- to 1242-nt (exon 3‒5) (Fig. 7, *A* and *B*). To investigate specific m6A motifs that m6A modification is dependent on, we constructed wildtype and two mutant *LINC00839* vectors (mutant-1: exon 3‒4; mutant-2: exon 5), in which the adenine residues in predicted m6A motifs of *LINC00839* were substituted by cytosine (A‒C mutant). The m6A methylated site mutations (wildtype, mutant 1, and mutant 2) of *LINC00839* were synthesized by Genscript and cloned into the pmir-GLO vectors to generate dual-luciferase vectors.

The dual-luciferase reporter constructs were cotransfected into HONE-1 or SUNE-1 cells. Each transfection group was cotransfected with Renilla plasmid. Luciferase activity was monitored by the Dual-Luciferase Reporter Assay Kit (Promega) according to the manufacturers’ instructions, and relative firefly luciferase activity was normalized to the Renilla luciferase activity.

### MeRIP

MeRIP was performed utilizing the Magna MeRIP m6A kit (Millipore) according to the manufacturer’s instructions. Briefly, anti-m6A antibody was bound to the magnetic beads at 4 °C overnight. About 300 mg of total RNA was fragmented using RNA fragmentation buffer and then incubated with the anti-m6A antibody, protease inhibitor, and RNase inhibitor. Targeted RNA was eluted and purified, which was analyzed by RT–qPCR. The specific primers are shown in Table S8.

### Animal experiments

All animal experiment procedures were approved by the Institutional Animal Care and Use Committee of Sun Yat-sen University Cancer Center (L102012022225L). Female BALB/c nude mice (6 weeks old, 18–20 g) were purchased from Beijing Vital River Lab Animal Technology. For xenograft growth model, 1 × 10^6^ SUNE-1 cells stably expressing scrambled or sh-*LINC00839* were inoculated subcutaneously into the axillas of nude mice. The tumor volumes were measured every 3 days. After 21 days, the mice were sacrificed. Simultaneously, the subcutaneous tumors were excised and weighed. For the lung metastatic colonization model, 1 × 10^6^ SUNE-1 cells stably expressing scrambled or sh-*LINC00839* were injected into the tail veins of nude mice. After 5 weeks, the mice were sacrificed, with their lung tissues dissected. All subcutaneous tumors and lung tissues were paraffin embedded and sectioned for subsequent analyses.

### Statistical analysis

All experiments were performed in triplicate. All statistical analyses were performed using GraphPad Prism, version 8.0 (GraphPad, Inc) or SPSS, version 22.0 (IBM Corporation). Data are presented as the mean ± SD. Student’s *t* test was used for comparisons of continuous variables, and Chi-squared test or Fisher’s exact test was used for categorical ones. One-way ANOVA followed by the Bonferroni test was performed for multiple comparisons. Receiver operating characteristic curve analysis was conducted to determine the optimal cutoff value for the stratification of high or low *LINC00839* expression groups. Kaplan–Meier analysis was performed, and differences of survival outcomes were compared using log-rank test. Univariate and multivariate Cox regression analyses were conducted to calculate hazard ratios and 95% confidence intervals. Correlations were performed by Pearson correlation analysis. A two-sided *p* value of 0.05 was considered statistically significant.

## Data availability

The RNA-Seq and ChIP-Seq data are accessible at the Gene Expression Omnibus repository (Gene Expression Omnibus accession number: GSE228725). The source data of this study are available from the corresponding author upon reasonable request.

## Ethics approval and consent to participate

The study protocol was approved by the institutional ethics committee of Sun Yat-sen University Cancer Center (grant no.: GZR2022-330), and the requirement for informed consent was waived given the retrospective nature of the study. All animal experiment procedures were approved by the Institutional Animal Care and Use Committee of Sun Yat-sen University Cancer Center (grant no.: L102012022225L).

## Supporting information

This article contains supporting information.

## Conflict of interest

The authors declare that they have no conflicts of interest with the contents of this article.
