# Supplementary material for: m6A-enriched lncRNA LINC00839 promotes tumor progression by enhancing TAF15-mediated transcription of amine oxidase AOC1 in nasopharyngeal carcinoma
Source: J Biol Chem. 2023 May 29;299(7):104873. doi: 10.1016/j.jbc.2023.104873 (PMC10302167; doi:10.1016/j.jbc.2023.104873)
Supplement: Supporting Tables S1–S8 [file mmc1.docx]

**Supplementary Tables: 8**

**Supplemental Table S1.** Relationship between *LINC00839* expression and clinicopathological characteristics of NPC patients (N = 214)

| **Characteristics** | **No. of patients** | **Expression of *LINC00839*** | | ***P* value** |
| --- | --- | --- | --- | --- |
|  |  | **Low, *n* (%)** | **High, *n* (%)** |  |
| **Age** |  |  |  | 0.22 |
| ≤45 | 95 | 52 (48.6) | 43 (40.2) |  |
| >45 | 119 | 55 (51.4) | 64 (59.8) |  |
| **Sex** |  |  |  | 0.63 |
| Male | 163 | 80 (74.8) | 83 (77.6) |  |
| Female | 51 | 27 (25.2) | 24 (22.4) |  |
| **VCA-IgA** |  |  |  | 0.07 |
| < 1:80 | 22 | 15 (14.0) | 7 (6.5) |  |
| ≥ 1:80 | 192 | 92 (86.0) | 100 (93.5) |  |
| **EA-IgA** |  |  |  | 0.59 |
| < 1:10 | 37 | 20 (18.7) | 17 (15.9) |  |
| ≥ 1:10 | 177 | 87 (81.3) | 90 (84.1) |  |
| **T Stage** |  |  |  | **0.04** |
| T1-T3 | 133 | 74 (69.2) | 59 (55.1) |  |
| T4 | 81 | 33 (30.8) | 48 (44.9) |  |
| **N Stage** |  |  |  | 0.28 |
| N0-N1 | 38 | 16 (15.0) | 22 (20.6) |  |
| N2-N3 | 176 | 91 (85.0) | 85 (79.4) |  |
| **TNM Stage** |  |  |  | 0.89 |
| III | 105 | 53 (49.5) | 52 (48.6) |  |
| IV | 109 | 54 (50.5) | 55 (51.4) |  |
| **Locoregional failure** | | | | **0.10** |
| Yes | 37 | 14 (13.1) | 23 (21.5) |  |
| No | 177 | 93 (86.9) | 84 (78.5) |  |
| **Distant metastasis** |  |  |  | **0.03** |
| Yes | 60 | 23 (21.5) | 37 (34.6) |  |
| No | 154 | 84 (78.5) | 70 (65.4) |  |
| **Tumor relapse** |  |  |  | **< 0.01** |
| Yes | 84 | 31 (29.0) | 53 (49.5) |  |
| No | 130 | 76 (71.0) | 54 (50.5) |  |
| **Death** |  |  |  | **0.01** |
| Yes | 80 | 31 (29.0) | 49 (45.8) |  |
| No | 134 | 76 (71.0) | 58 (54.2) |  |

VCA-IgA: viral capsid antigen immunoglobulin A; EA-IgA: early antigen immunoglobulin A; HR, hazard ratio. All patients were restaged according to the 8^th^ edition of the AJCC Cancer Staging Manual. Bold values indicate *P*< 0.05; *P* value is determined by χ^2^and Fisher’s exact tests.

**Supplemental Table S2.** Cox regression analysis of clinical variables contributing to overall survival, disease-free survival and distant metastasis-free survival in NPC patients (n = 214)

|  | **Univariate analysis** | | |  | **Multivariate analysis** | | |
| --- | --- | --- | --- | --- | --- | --- | --- |
| **Variable** | **HR** | **95%CI** | ***P-*value** |  | **HR** | **95%CI** | ***P-*value** |
| **Overall survival** |  |  | |  |  |  |  |
| *LINC00839* (High *vs.* Low) | 1.89 | 1.20–2.98 | **< 0.01** |  | 1.85 | 1.17–2.92 | **< 0.01** |
| TNM stage (IV *vs.* III) | 1.82 | 1.16–2.86 | **0.01** |  | 1.89 | 1.20–2.97 | **< 0.01** |
| Sex (Male *vs.* Female) | 1.58 | 1.00–2.50 | **0.05** |  | 1.39 | 0.87–2.21 | 0.17 |
| Age (>45 years *vs.* ≤45 years) | 2.30 | 1.22–4.35 | **0.01** |  | 2.21 | 1.16–4.21 | **0.02** |
| VCA-IgA (≥ 1:80 *vs.* < 1:80) | 1.12 | 0.54–2.33 | 0.76 |  |  |  |  |
| EA-IgA (≥ 1:10 *vs.* < 1:10) | 1.17 | 0.65–2.12 | 0.60 |  |  |  |  |
| **Disease-free survival** |  | | |  |  |  |  |
| *LINC00839* (High *vs.* Low) | 2.09 | 1.34–3.27 | **< 0.01** |  | 1.87 | 1.20–2.90 | **< 0.01** |
| TNM stage (IV *vs.* III) | 1.86 | 1.20–2.89 | **< 0.01** |  | 2.11 | 1.35–3.28 | **< 0.01** |
| Sex (Male *vs.* Female) | 1.13 | 0.73–1.75 | 0.57 |  |  |  |  |
| Age (>45 years *vs.* ≤45 years) | 1.68 | 0.96–2.95 | 0.07 |  |  |  |  |
| VCA-IgA (≥ 1:80 *vs.* < 1:80) | 1.14 | 0.55–2.37 | 0.72 |  |  |  |  |
| EA-IgA (≥ 1:10 *vs.* < 1:10) | 1.27 | 0.70–2.29 | 0.44 |  |  |  |  |
| **Distant metastasis-free survival** |  | | |  |  |  |  |
| *LINC00839* (High *vs.* Low) | 1.88 | 1.12–3.17 | **0.02** |  | 1.89 | 1.12–3.18 | **0.02** |
| TNM stage (IV *vs.* III) | 2.44 | 1.42–4.21 | **< 0.01** |  | 2.45 | 1.42–4.22 | **< 0.01** |
| Sex (Male *vs.* Female) | 1.21 | 0.72–2.03 | 0.47 |  |  |  |  |
| Age (>45 years *vs.* ≤45 years) | 1.75 | 0.89–3.45 | 0.11 |  |  |  |  |
| VCA-IgA (≥ 1:80 *vs.* < 1:80) | 1.76 | 0.64–4.85 | 0.28 |  |  |  |  |
| EA-IgA (≥ 1:10 *vs.*< 1:10) | 2.15 | 0.93–5.01 | 0.08 |  |  |  |  |

VCA-IgA: viral capsid antigen immunoglobulin A; EA-IgA: early antigen immunoglobulin A; HR, hazard ratio. All patients were restaged according to the 8^th^ edition of the AJCC Cancer Staging Manual. Bold values indicate *P*<0.05, *P* value is determined by Cox regression analysis.

**Supplemental Table S3.** RT-qPCR primers used in this study

| **Gene** | **Sequence (5’ to 3’)** |
| --- | --- |
| *LINC00839*-F | TGACGCCCACTGCTAGAAGT |
| *LINC00839*-R | TCTGGCCGATTATCTGCAGC |
| *LINC00839*-S-F  (for survival analysis) | GGAAACTGCCCATACGGACCTAC |
| *LINC00839*-S-R  (for survival analysis) | TCCTGGAACAATCTGGGCCTGAA |
| *TAF15*-F | GATTCTGGAAGTTACGGTCAGTC |
| *TAF15*-R | AGCTTTGTGATGCTTGTCCATAG |
| *IGF2BP1*-F | GCGGCCAGTTCTTGGTCAA |
| *IGF2BP1*-R | TTGGGCACCGAATGTTCAATC |
| *AOC1*-F | CCTAAGCAACCAAGAGCTGAA |
| *AOC1*-R | CGGTGACATTGGGATGCTCC |
| *VIRMA*-F | TGACCTTGCCTCACCAACTGCA |
| *VIRMA*-R | AGCAACCTGGTGGTTTGGCTAG |
| *GAPDH*-F | TGATGACATCAAGAAGGTGG |
| *GAPDH*-R | TTGTCATACCAGGAAATGAGC |
| *GAPDH*-S-F  (for survival analysis) | GACAGTCAGCCGCATCTTCTTTTGC |
| *GAPDH*-S-R  (for survival analysis) | GCGCCCAATACGACCAAATCCGTT |

**Supplemental Table S4.** siRNA sequence used in this study

| **Name** | **Sequence (5’ to 3’)** |
| --- | --- |
| si-*LINC00839*-1#-F | GCGGAUGACCAGCUACAAUTT |
| si-*LINC00839*-1#-R | AUUGUAGCUGGUCAUCCGCTT |
| si-*LINC00839*-2#-F | GCCUUAAACUGACCAUGAATT |
| si-*LINC00839*-2#-R | UUCAUGGUCAGUUUAAGGCTT |
| si-*TAF15*-1#-F | GGACAGAACUACAGCGGUUTT |
| si-*TAF15*-1#-R | AACCGCUGUAGUUCUGUCCTT |
| si-*TAF15*-2#-F | GGAAUCAUCAGGAAGCCAATT |
| si-*TAF15*-2#-R | UUGGCUUCCUGAUGAUUCCTT |
| si-*AOC1*-1#-F | GGAUAAAGGUGAAAGGCAUTT |
| si-*AOC1*-1#-R | AUGCCUUUCACCUUUAUCCTT |
| si-*AOC1*-2#-F | GCCGCAGUUGGCUUAUCAUTT |
| si-*AOC1*-2#-R | AUGAUAAGCCAACUGCGGCTT |
| si-*IGF2BP1*-1#-F | GGCUCAGUAUGGUACAGUATT |
| si-*IGF2BP1*-1#-R | UACUGUACCAUACUGAGCCTT |
| si-*IGF2BP1*-2#-F | CAUGCCGCAUGAUUCUUGATT |
| si-*IGF2BP1*-2#-R | UCAAGAAUCAUGCGGCAUGTT |
| si-*VIRMA*-1#-F | CCAUCAUCUUUAGACCUAATT |
| si-*VIRMA*-1#-R | UUAGGUCUAAAGAUGAUGGTT |
| si-*VIRMA*-2#-F | GCUGAUCACGUAUCAUCUUTT |
| si-*VIRMA*-2#-R | AAGAUGAUACGUGAUCAGCTT |

**Supplemental Table S5.** Primers used for shRNA plasmid construction

| **Name** | **Sequence (5’ to 3’)** |
| --- | --- |
| sh-*LINC00839*-1#-F | CCGGGCCCAACATATTCCTTTAATTCTCGAGAATTAAAGGAATATGTTGGGCTTTTTG |
| sh-*LINC00839*-1#-R | AATTCAAAAAGCCCAACATATTCCTTTAATTCTCGAGAATTAAAGGAATATGTTGGGC |
| sh-*LINC00839*-2#-F | CCGGCCAGCTACAATGAGTAATCTACTCGAGTAGATTACTCATTGTAGCTGGTTTTTG |
| sh-*LINC00839*-2#-R | AATTCAAAAACCAGCTACAATGAGTAATCTACTCGAGTAGATTACTCATTGTAGCTGG |

**Supplemental Table S6.** Mass spectrometry results for biotinylated *LINC00839* RNA pulldown experiments in the antisense group (negative control).

| **Protein** | **Score** |
| --- | --- |
| Serum albumin, ALB | 285 |
| Keratin, type I cytoskeletal 9, KRT9 | 235 |
| Keratin, type I cytoskeletal 14, KRT14 | 234 |
| Keratin, type II cytoskeletal 6B, KRT6B | 159 |
| HCG2039812, KRT6A | 138 |
| HCG15971, PS1TP5BP1 | 79 |
| Keratin 18, KRT18 | 73 |
| T cell receptor alpha joining 56, TRAJ56 | 52 |
| Dynein heavy chain 12, DNAH12 | 27 |

**Supplemental Table S7.** Mass spectrometry results for biotinylated *LINC00839* RNA pulldown experiments in the sense group. The **bold portion** shows the proteins that overlap antisense group.

| **Protein** | **Score** |
| --- | --- |
| Probable ATP-dependent RNA helicase, DDX17 | 254 |
| **Keratin, type I cytoskeletal 9, KRT9** | **235** |
| Polypyrimidine tract-binding protein 1, PTBP1 | 217 |
| 60S ribosomal protein L18a, RPL18A | 118 |
| Insulin-like growth factor 2 mRNA binding protein 1, IGF2BP1 | 101 |
| Y-box-binding protein 1, YBX1 | 89 |
| Serine/arginine-rich-splicing factor 1, SRSF1 | 86 |
| TATA-binding protein-associated factor, TAF15 | 31 |
| RNA-binding protein 39, RBM39 | 15 |

**Supplemental Table S8.** Primers sequence used for ChIP-qPCR and MeRIP-qPCR assays

| **Name** | **Sequence (5’ to 3’)** |
| --- | --- |
| *LINC00839*-m6A-F1  (for MeRIP-qPCR) | TGCCTTTGCTTTTATCGCTT |
| *LINC00839*-m6A-R1  (for MeRIP-qPCR) | CATTCGTGCAGAAATCCTGT |
| *LINC00839*-m6A-F2  (for MeRIP-qPCR) | TGCAGCCATGTCAAGACTGA |
| *LINC00839*-m6A-R2  (for MeRIP-qPCR) | CAGATTTTCTTCCGCCATC |
| *AOC1*-CHIP-F1  (for CHIP-qPCR) | GCGTCTTTACTGTCATGCCAT |
| *AOC1*-CHIP-R1  (for CHIP-qPCR) | ACCCCTCTATTCTGAGAACCC |
| *AOC1*-CHIP-F2  (for CHIP-qPCR) | CCTCAACGTAAGTCCTAGGCCAA |
| *AOC1*-CHIP-R2  (for CHIP-qPCR) | GCATGACAGTAAAGACGCACT |
